# Origin of Li^+^ Solvation Ability of Electrolyte Solvent: Ring Strain

**DOI:** 10.3390/ma16216995

**Published:** 2023-10-31

**Authors:** Jihoon Choi, Kyoung-Hee Shin, Young-Kyu Han

**Affiliations:** 1 Department of Energy and Materials Engineering, Advanced Energy and Electronic Materials Research Center, Dongguk University-Seoul, Seoul 04620, Republic of Korea; chchc2059@gmail.com; 2 ESS Laboratory, Korea Institute of Energy Research, 102 Gajeong-ro, Daejeon 34129, Republic of Korea; khshin@kier.re.kr

**Keywords:** lithium metal battery, solvent–ion interaction, electrolyte, ring strain, first-principles calculation

## Abstract

Developing new organic solvents to support the use of Li metal anodes in secondary batteries is an area of great interest. In particular, research is actively underway to improve battery performance by introducing fluorine to ether solvents, as these are highly compatible with Li metal anodes because fluorine imparts high oxidative stability and relatively low Li-ion solvation ability. However, theoretical analysis of the solvation ability of organic solvents mostly focuses on the electron-withdrawing capability of fluorine. Herein, we analyze the effect of the structural characteristics of solvents on their Li^+^ ion solvation ability from a computational chemistry perspective. We reveal that the structural constraints imposed on the oxygen binding sites in solvent molecules vary depending on the structural characteristics of the N-membered ring formed by the interaction between the organic solvent and Li^+^ ions and the internal ring containing the oxygen binding sites. We demonstrate that the structural strain of the organic solvents has a comparable effect on Li^+^ solvation ability seen for the electrical properties of fluorine elements. This work emphasizes the importance of understanding the structural characteristics and strain when attempting to understand the interactions between solvents and metal cations and effectively control the solvation ability of solvents.

## 1. Introduction

The use of Li metal as the anode material in Li-ion batteries is a highly active research area due to the high theoretical specific capacity (3860 mAh g^−1^) and the low negative electrochemical potential (−3.04 V vs. the standard hydrogen electrode) of this metal [1,2,3]. However, an irreversible Li plating/stripping process leads to the formation of Li dendrites that cause short circuits and pose a significant safety risk [4,5,6]. Quantitative analyses using accelerating rate calorimeters have demonstrated that the thermal stability of Li-metal batteries decreases significantly due to the formation of Li dendrites after repeated cycles [7,8,9]. Moreover, the non-uniform solid electrolyte interphase (SEI) that forms on the Li metal surface results in continuous side reactions that reduce the battery’s Coulombic efficiency [10,11,12,13]. Various strategies have been invoked to address these issues, but one of the most effective approaches has been electrolyte engineering. The electrolyte engineering approach allows adjustment of the characteristics and properties of the electrolyte, thereby reducing its reactivity with Li metal and improving the compatibility with high-voltage cathodes [14,15,16,17,18,19,20]. 

Commercially available carbonate electrolytes exhibit high ionic conductivity and good compatibility with graphite anodes when combined with LiPF_6_, which forms a stable SEI layer [21,22]. However, the properties of carbonate electrolytes cause them to undergo oxidative decomposition at 4.5 V, leading to decreased oxidative stability and cycling performance, as well as inducing an inhomogeneous SEI layer composed of amorphous organic polymers that degrade interfacial performance at the anode [23,24,25,26]. These drawbacks are further exacerbated in the case of Li-metal anodes, as they promote the formation of Li dendrites through side reactions. By contrast, ether-based electrolytes can form a relatively stable SEI layer on the Li-anode surface and suppress side reactions and Li dendrite formation [21,27,28,29]. However, molecules such as 1,2-dimethoxyethane (DME) and 1,3-dioxolane (DOL), which are commonly used as ether solvents, exhibit low oxidative stability at high voltages [30,31,32]. Many studies are presently being conducted to overcome the drawbacks of these ether solvents while maintaining their advantages. One representative strategy is the introduction of fluorine atoms to ether solvents to improve oxidation stability, and the replacement of salts with lithium bis(fluorosulfonyl)imide (LiFSI) to form a more stable anion-derived SEI layer with superior performance [2,19,22,25,26,33,34,35,36,37,38].

In an electrolyte, Li^+^ is solvated by the ether-based solvents and FSI anions to form a Li^+^ solvation sheath [39,40]. Components that participate mainly in the solvation sheath can diffuse to the anode along with Li^+^ ion and contribute more easily to SEI formation on the anode surface [36,41]. When FSI anions participate mainly in the solvation sheath, an anion-derived SEI can improve the distribution of Li^+^ within the interphase and increase Coulombic efficiency [35,37,42,43]. Formation of an anion-derived SEI requires an appropriately reduction in the solvation ability of the electrolyte solvents, as well as an effective and systematic strategy for controlling the solvation ability of the solvents. The main strategy used for this is to incorporate fluorinated solvents, as these have low solvation ability through the electron-withdrawing effect of fluorine atoms. 

**Figure 1:**
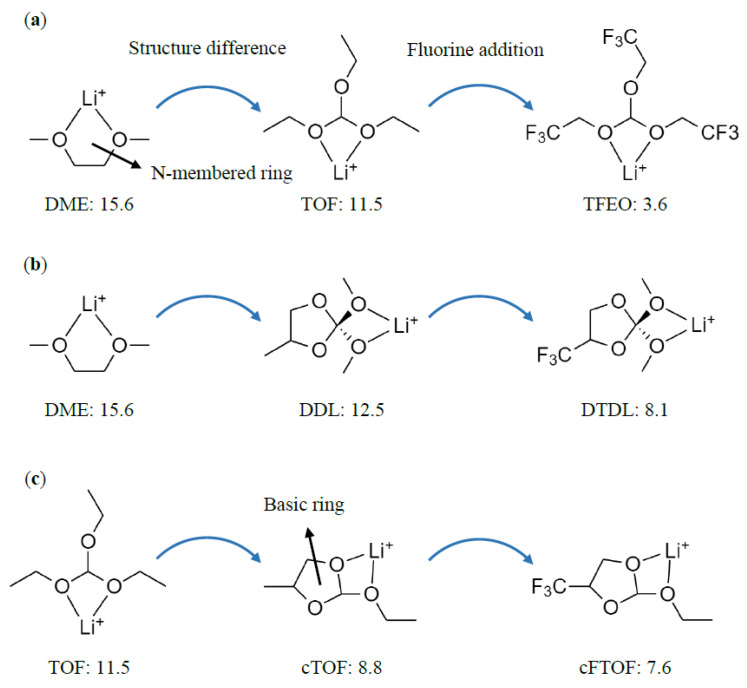
Calculated Li^+^ binding energies of the solvent molecules (**a**) DME, TOF, and TFEO, (**b**) DME, DDL, and DTDL, and (**c**) TOF, cTOF, and cFTOF. Units are in kcal/mol.

Many studies have modified the composition of the solvation sheath by exploiting the electron-withdrawing effect of fluorine atoms. Recently, Cao et al. used tris(2,2,2-trifluoroethyl)orthoformate (TFEO) as a cosolvent with DME to reduce the solvent’s solvation ability [38]. As a result, more FSI anions participated in the solvation sheath, leading to the formation of an anion-derived SEI with excellent performance. When TFEO was added to DME as a cosolvent, ionic conductivity decreased by 83%. Zhao et al. adjusted the solvation sheath using a fluorinated ether—namely, 2,2-dimethoxy-4-(trifluoromethyl)-1,3-dioxolane (DTDL) [22]. They then used the electron-withdrawing −CF_3_ functional group to ensure the solvent’s oxidation stability and appropriately reduced its solvation ability, thereby allowing more FSI anions to participate in the solvation sheath. When DTDL was used, ionic conductivity decreased by 93%. Furthermore, an upfield shift was observed in the ^7^Li and ^19^F NMR spectra of DTDL compared to DME, indicating that the solvation ability of DTDL was lower than that of DME. Zhou et al. compared the performance of electrolytes using triethyl orthoformate (TOF) and 2-ethoxy-4-(trifluoromethyl)-1,3-dioxolane (cFTOF) as single solvents in high-voltage Li-metal batteries after converting TOF to cFTOF through a one-pot reaction [33]. Due to the presence of −CF_3_ moieties, more FSI anions participated in the solvation sheath formed with cFTOF than with TOF. When cFTOF was used as a solvent instead of TOF, ionic conductivity decreased by 87%. An upfield shift in the ^7^Li and ^19^F NMR spectra was observed in cFTOF compared to TOF, indicating stronger interaction between the FSI anions and Li^+^ due to the lower solvation ability of cFTOF. Although various studies have introduced new organic solvents with appropriately modified solvation ability and have shown significant performance improvement, a lack of understanding remains regarding how the unique characteristics of organic solvents affect their solvation ability and how best to design new solvents with superior performance. Most studies only utilize the electron-withdrawing effect of fluorine atoms to analyze the solvation ability, and theoretical interpretations of how the unique characteristics of organic solvents affect the solvation ability are very scarce. In this study, we have systematically investigated how the structural characteristics of TFEO, DTDL, and cFTOF affect their solvation ability and promote the formation of an anion-derived SEI. To allow consideration of only the structural characteristics of the molecules, we compared TOF, DDL, and cTOF, which are molecules in which all the fluorine atoms in TFEO, DTDL, and cFTOF are replaced with hydrogen atoms, using DME and TOF (see Figure 1). The optimized structures of the considered molecules are shown in Figure S1.

Our investigation revealed that the structural strain of the Li^+^−solvent complex is closely connected to the size of the N-membered ring (the ring formed by the interaction between Li^+^ and solvent) or the basic ring (the ring containing the oxygen-binding site present in the solvent itself). If the structural strain is high, the Li^+^ coordinated ring structures become unstable, leading to a decrease in the Li^+^ binding energy. The 4-membered ring of TOF and DDL forms a higher strain structure than the 5-membered ring of DME, resulting in lower Li^+^ binding energies for TOF and DDL. The cTOF molecule, which has a 5-membered basic ring containing the oxygen binding site, experiences significant structural strain, while the TOF molecule, which does not have a basic ring, is not subjected to structural strain, leading to a relatively low Li^+^ binding energy for cTOF. The introduction of fluorine atoms to TOF, DDL, and cTOF, resulting in TFEO, DTDL, and cFTOF, respectively, leads to a further decrease in solvent ability due to the electrical properties of the fluorine atoms. We clearly show here that the structural characteristics, as well as the electrical properties, are important factors that determine the solvation ability of the solvents.

## 2. Methods

A computational study based on Kohn−Sham density functional theory was performed to calculate the structural and electrical properties of organic molecules [44,45,46]. The Kohn–Sham equation is the non-interacting Schrödinger equation of a fictitious system of non-interacting particles (typically electrons) that generate the same density as any given system of interacting particles [47,48]. All molecular structures were optimized using the Gaussian16 software package [49] with the B3LYP functional [50,51] and 6-311++G(d,p) basis set [52]. A DFT functional for calculating energy and a basis set for describing electronic behavior are required. In this study, a diffuse function augmented the triple-zeta quality of the Pople group basis set along with B3LYP, the most widely used hybrid functional in molecular calculations. The Gaussian program is primarily used for calculating finite size molecules with Gaussian-type atomic orbitals. This functional is widely used in metal-ion battery systems and provides accurate descriptions of structures and electrostatic interactions [53,54,55]. The polarizable continuum model (PCM), which is one of the self-consistent reactive field methodologies, was used to account for the bulk solvent effects on the molecular structures and electrical properties [56,57]. The PCM model by Tomasi and coworkers is one of the most frequently used continuum solvation methods using an implicit solvent description. Considering that the organic molecules used in the calculations are ether-based, the dielectric constant ε = 7.40 of tetrahydrofuran was adopted, as it approximates the dielectric constant ε = 7.30 of DME [58,59,60,61,62]. Natural bond orbital analysis was conducted to investigate oxygen–lithium interactions [63,64]. Topological properties at the Li^+^−O bond were studied by combining Gaussian16 with the Multiwfn 3.8 program [65]. For all complexes, the binding energy (E_b_) between Li^+^ and explicit solvent molecules was defined by Equation (1).

(1) $$E_{b}=-[E\left({Li}^{+}solvent\;complex\right)-E\left({Li}^{+}\right)-E(solvent)]$$

where E(Li^+^–solvent complex), E(Li^+^), and E(solvent) represent the energies of the lithium ion–solvent complex, lithium cation, and solvent molecules, respectively. Although many more variables are involved in the reactions within the actual secondary battery, the first-principles calculation that we chose is the most suitable and widely used method for studying the attraction between metal atoms and organic molecules, and various studies using this approach have been conducted [66,67,68,69,70]. We calculated the Li^+^ binding energy using the B3LYP-D3 [71], M05-2X [72], PBE [73], and M06-L [74] functionals, and compared them to the B3LYP results. To investigate the binding energy in the Li^+^ solvation sheath, we performed calculations (i) for the case of two solvents, where each interacts in a two-coordinate manner with Li^+^ ions and (ii) for the case of one FSI anion and one solvent, where each interacts with Li^+^ ions in a two-coordinate way.

Three reaction energies were considered, as depicted in Figure S2, considering that Li^+^ can attach to a solvent molecule either at one-coordinate or two-coordinate. One-coordinate refers to the state in which the Li cation interacts strongly with one oxygen atom in the solvent while having very weak interaction with another oxygen atom. Two-coordinate describes the state in which the Li cation interacts strongly with two oxygen atoms in the solvent molecules. The one-coordinate binding energy (E_b1_) is the reaction energy when Li^+^ attaches to a solvent molecule in the one coordinate, while the ring formation energy (E_R_) is the reaction energy when Li^+^ attaches to a solvent molecule from the one-coordinate to the two-coordinate. The sum of E_b1_ and E_R_ gives the two-coordinate binding energy of Li^+^ (E_b2_).

We evaluated the ring strain energy (ΔE_RS_) to take into account the structural characteristics of the molecule, considering that the ring formed by the Li^+^ two-coordinate binding has unique structural characteristics of a metal cation−organic molecule complex [75,76,77,78,79,80]. The ring strain energy (ΔE_RS_) is defined by Equation (2). E_N-cyclo_ and E_N-acyclo_ represent the energies of the N-membered ring structure and the corresponding open-chain structure, respectively. The ΔE_RS_ value was defined based on the case of N = 6, because it is well known that the ring strain is the smallest when N = 6, and this approach is widely used to explain ring strain [75,76,77].

(2) $$∆E_{RS}=\left(E_{N-cyclo}-E_{N-acyclo}\right)-\left(E_{6-cyclo}-E_{6-acyclo}\right)$$

## 3. Results and Discussion

TFEO, DTDL, and cFTOF are organic solvents that exhibit significantly improved SEI formation and superior electrochemical performance compared to the conventional solvents DME and TOF, due to the introduction of electronegative fluorine elements [22,33,38]. We note that the fluorinated solvents are structurally different from DME and TOF; therefore, analyzing how their structural characteristics affect their solvation abilities becomes important. To assess the contribution of structural characteristics to the solvation abilities of these solvents, TOF, DDL, and cTOF (which are molecules in which all fluorine atoms of TFEO, DTDL, and cFTOF are replaced by hydrogen atoms) were considered for comparison with DME and TOF (see Figure 1). The optimized structures of the molecules coordinated to Li^+^ are shown in Figure S3. According to the comparison of Li^+^ binding energies in Figure 1, the Li^+^ binding energy is the lowest for TFEO, DTDL, and cFTOF, due to the contribution of the fluorine atoms. Interestingly, even in TOF, DDL, and cTOF, which show only structural differences and no contribution of fluorines, a significantly reduced Li^+^ binding energy was still observed. This indicates that the decrease in Li^+^ binding energy due to structural differences is as significant as the contribution of the fluorines. In other words, the Li^+^ binding energy is significantly reduced and the solvation ability is decreased not only due to the electrostatic contribution of fluorines but also due to the structural characteristics of the solvent molecules. The comparison of TOF, cTOF, and cFTOF, in particular, confirms that the influence of the structural difference is even greater than the electronic contribution from fluorines.

### 3.1. Structural Effects of N-Membered Rings on Solvation Ability

**Figure 2:**
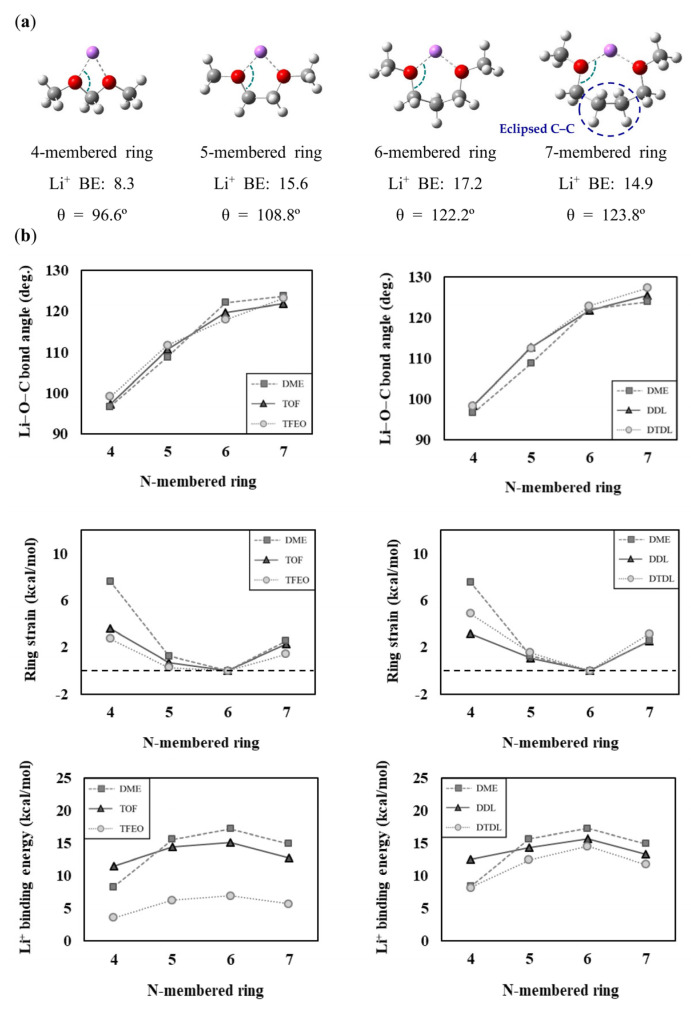
(**a**) Calculated Li^+^ binding energies (BE; in kcal/mol), Li−O−C bond angles (θ in degree) of 4- to 7-membered rings for DME. (**b**) Comparison of the Li−O−C bond angles, ring strain energies, and Li^+^ binding energies according to the N-membered ring. The dashed lines represent the zero point on the y-axis.

Since the size of the N-membered ring formed by the binding with Li^+^ differs in DME (N = 5) compared to TOF and DDL (N = 4), we analyzed how the size of the N-membered ring might affect the solvation ability. First, we checked how the structural strain changes according to the size of the N-membered ring by making four- to seven-membered ring structures based on DME, TOF, TFEO, DDL, and DTDL molecules, as shown in Figure S4. Based on these structures, we calculated the Li−O−C bond angle, ring strain energy, and Li^+^ binding energy, and presented them graphically in Figure 2. The corresponding numerical values are presented in Table S1. The Li−O−C bond angle is the internal angle of the N-membered ring, as shown in Figure 2a. As shown in Figure 2b, as the ring size increases from four-membered to six-membered rings, the Li−O−C bond angle increases (97.0°→122.2°) for DME, resulting in a decrease in structural strain. Although the Li−O−C bond angle also slightly increases in the six-membered ring (123.8°) for DME, the increase in torsional strain due to the formation of eclipsed C−C bonds, as shown in Figure 2a, results in an increased structural strain compared to the six-membered ring [81]. The seven-membered ring is structurally constrained to represent an eclipsed C–C bond, resulting in strong repulsive interactions (1) between the two nearby hydrogens of the occluded C–C bond and (2) of gauche conformations of two –OCH_2_CH_2_CH_2_– bonds. Therefore, the six-membered ring has the lowest ring strain and the highest Li^+^ binding energy. Conversely, the four-membered ring has the highest ring strain and the lowest Li^+^ binding energy. This is why TOF and DDL, which form four-membered rings by binding to Li^+^, have relatively high ring strain and low Li^+^ binding energy compared to DME, which forms a five-membered ring.

To obtain a better understanding of the effect of ring strain on the interaction between Li^+^ and organic molecules, we also presented the one-coordinate and two-coordinate Li^+^ binding energies, along with the ring formation energies according to N values of each solvent molecule in Figure S5. The calculated values are summarized in Table S2. The DME, TOF, TFEO, DDL, and DTDL molecules showed no significant differences in the E_b1_ according to N values. Except for TFEO, which has a large electron-withdrawing effect due to its substitution with nine fluorine atoms, no significant differences were also detected for the E_b1_ between the different solvent molecules. However, the E_b2_, which is influenced by ring strain, varied significantly depending on the N values and different solvent molecules. Adding the ring formation energy to the E_b1_ results in the E_b2_, and if the ring strain is high, the ring formation energy decreases, leading to a low E_b2_. This suggests that ring strain contributes significantly to the solvation ability of solvents when the binding state with Li^+^ is transformed from a one-coordinate to a two-coordinate type.

**Figure 3:**
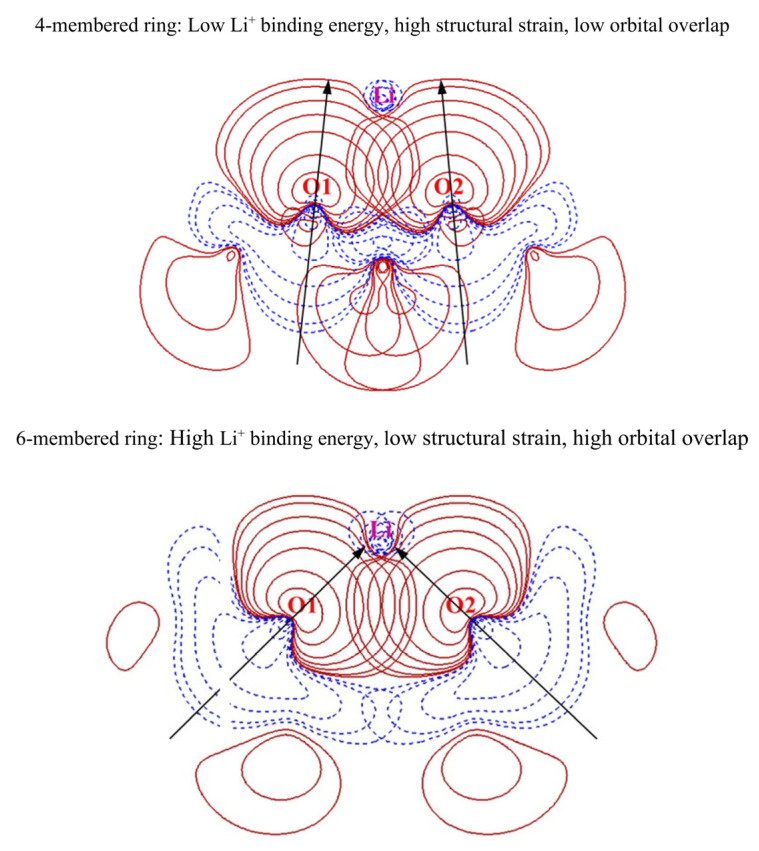
Contour plots of oxygen lone pair orbitals and lithium antibonding lone pair orbitals of 4- or 6-membered rings. The black arrows represent the direction of the lone pair orbitals of the oxygen binding sites.

To interpret the contribution of the N-membered ring size to the interaction between organic molecules and lithium cations, we plotted contour plots of the lone pair (LP) orbitals of the oxygen binding site and the antibonding lone pair (LP*) orbitals of Li^+^ in Figure 3. In the contour plot, the red lines correspond to the regions where the electron density shows positive values, indicating the presence of electrons in that region. On the other hand, the blue lines represent the regions where the electron density has negative values, indicating the absence of electrons in that region. Furthermore, the contour scale is displayed in a sequence starting from the outermost region with values of 0.004, 0.008, 0.020, 0.040, 0.080, 0.200, and 0.400. The LP orbitals of the oxygen binding site are widely known to play a significant role in the orbital interaction with metal cations [53,54,82,83,84,85,86,87,88,89,90]. To form a two-coordinate bond between Li^+^ and the organic solvent, the lobes of the LP orbitals of both oxygen atoms should be oriented toward Li^+^. However, if the structural strain of the N-membered ring is high, the LP orbitals of both oxygen atoms have difficulty in properly orienting toward the position of Li^+^. The contour plots in Figure 3 show that the four-membered ring with high structural strain has an oxygen−lithium bond direction that is misaligned with the oxygen’s LP lobe. By contrast, the six-membered ring with low structural strain has an oxygen−lithium bond direction that is well aligned with the oxygen’s LP lobe. Furthermore, examination of the contour plots of the orbital wave function reveals that the overlap between the oxygen’s LP orbital and the lithium’s LP* orbital is low in the four-membered ring, while the overlap is high in the six-membered ring. As shown in Figure S6, based on the second-order perturbation theory [91] through NBO analysis, the orbital interaction between oxygen and lithium is stronger in the six-membered ring than in the four-membered ring. This suggests that, in N-membered rings with high structural strain, the overlap between the oxygen LP and lithium LP* orbitals is low, indicating weak Li^+^ solvation ability.

### 3.2. Structural Effects of Basic Rings on Solvation Ability

**Figure 4:**
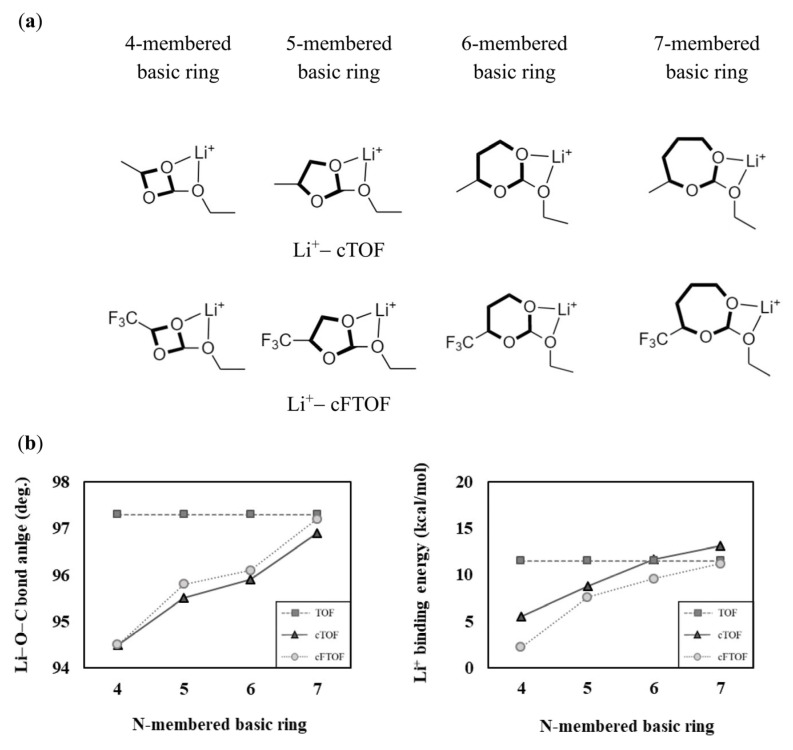
(**a**) Li^+^-coordinated structures of 4- to 7-membered 2D basic ring structures based on cTOF and cFTOF. (**b**) Comparison of the Li−O−C bond angles and Li^+^ binding energies according to the N-membered basic ring. The TOF values without a basic ring are presented for comparison.

In the cases of TOF, cTOF, and cFTOF, due to the structural differences between TOF and cTOF, they exhibit different solvation abilities. However, since they have the same sized N-membered rings, we cannot interpret the difference in solvation ability using the approach described in Section 3.1. We noted structural differences in the oxygen binding site present in the chain of TOF and in the basic ring of cTOF, cFTOF. To investigate how the structural characteristics and solvation ability of the molecules change with the size of the basic ring, we calculated the Li−O−C bond angle and the Li^+^ binding energy for four- to seven-membered basic rings of cTOF and cFTOF shown in Figure 4a, and presented the results in Figure 4b. The corresponding numerical values are presented in Table S3. When the oxygen binding site is included in a seven-membered basic ring of cTOF (96.9°), the Li−O−C bond angle becomes close to that of TOF (97.3°), a chain molecule without a ring, so we can see that the effect of the basic ring size on the interaction with Li^+^ decreases as the size of the basic ring increases. Conversely, as the size of the basic ring decreases, the Li−O−C bond angle gradually decreases from 96.9° (for the seven-membered basic ring) to 94.5° (for the four-membered basic ring) for cTOF, resulting in a continuous increase in structural strain due to the angle, leading to a decrease in Li^+^ binding energy. In other words, cTOF and cFTOF, which have five-membered basic rings, show lower Li−O−C bond angles compared to the chain molecule TOF, indicating that cTOF and cFTOF have a relatively high strain due to the basic ring and can be interpreted as having lower Li^+^ binding energy than TOF. If the oxygen binding site belongs to a small-sized basic ring, the structural strain increases due to the limited internal angle of the basic ring. Therefore, the solvation ability decreases because the effective orbital interaction between oxygen and Li^+^ becomes difficult.

**Figure 5:**
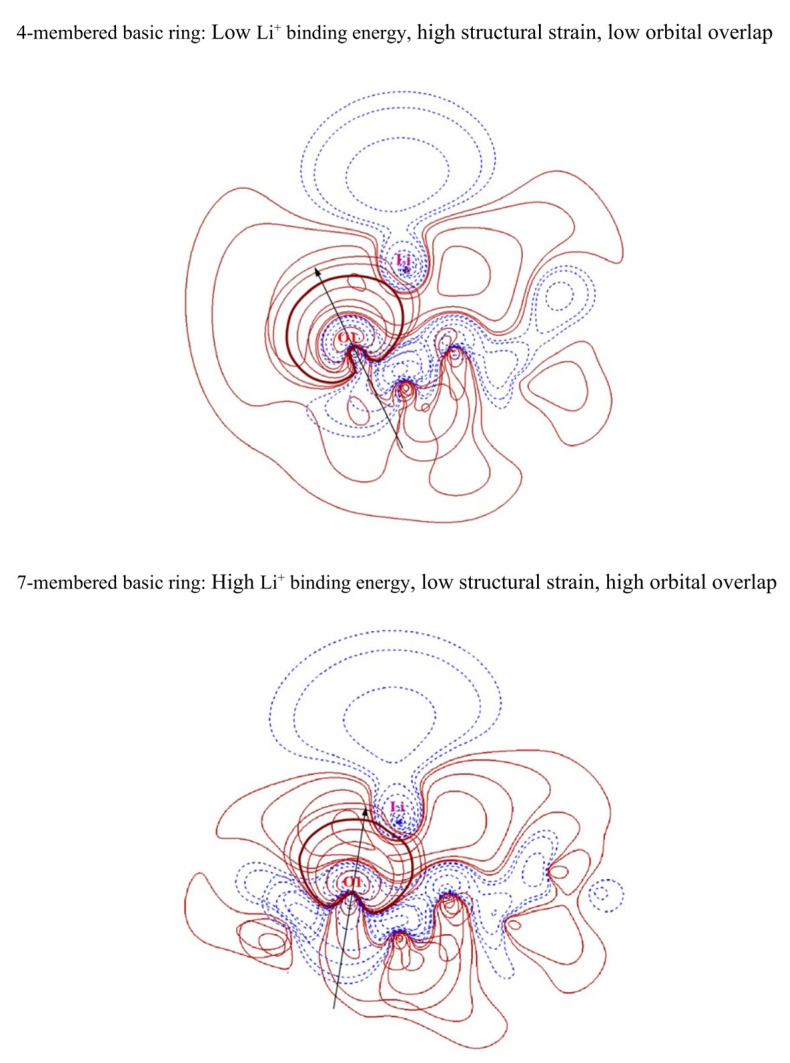
Contour plots of oxygen lone pair orbitals and lithium antibonding lone pair orbitals of 4- or 7-membered basic rings. The black arrows represent the direction of lone pair orbitals of the oxygen binding sites. The bold red lines are the 0.020 isovalue lines of oxygen lone pair orbitals.

To interpret the reason why the size of N-membered basic rings contributes to the interaction between solvent molecules and lithium cations, we present contour plots of the LP orbitals of the oxygen binding site and the LP* orbitals of lithium in Figure 5. In Figure 5, O1 is the oxygen binding site in the basic ring. From the contour plots, we observed that, in the four-membered basic ring, which has high structural strain, the direction of the oxygen−lithium bond largely deviates from the direction of the oxygen’s LP lobe, while in the seven-membered basic ring, which has low structural strain, the direction of the oxygen−lithium bond aligns relatively well with the direction of the oxygen’s LP lobe. Furthermore, based on the contour plots of the orbital wave function, we see that the overlap between the LP orbital of oxygen and the LP* orbital of lithium is low in the four-membered basic ring, while the overlap is relatively high in the seven-membered basic ring. As seen in Figure S7, the direction of the oxygen–lithium bonds in TOF aligns well with the direction of the oxygen’s LP lobe, and the overlap between the LP orbital of oxygen and the LP* orbital of lithium of TOF is higher than that in the cTOF and cFTOF cases. In the second-order perturbation theory shown in Figure S8, the orbital interaction between oxygen and lithium is stronger in the seven-membered basic ring than in the four-membered basic ring. This suggests that, in N-membered basic rings with high structural strain, the overlap between the oxygen LP and lithium LP* orbitals is low, indicating weak Li^+^ solvation ability.

### 3.3. DFT Functional Dependence and Consideration of Solvent−Solvent and Solvent−Salt Interactions within the Solvation Sheath

We assessed the influence of DFT functionals on the Li^+^ binding energies with solvents by evaluating the Li^+^ binding energies using several functionals (B3LYP-D3, M05-2X, PBE, and M06-L). As shown in Figure S9, although the binding energies quantitatively depend on the choice of functional, the trends for the N values were confirmed to be consistent with the B3LYP results: As the ring size increases from a four-membered to a six-membered ring, the ring strain decreases, resulting in an increase in Li^+^ binding energy. However, in the case of a seven-membered ring, torsional strain leads to a decrease in the binding energy. In an N-membered basic ring, as the value of N increases, the structural strain decreases, leading to an increase in Li^+^ binding energy.

**Figure 6:**
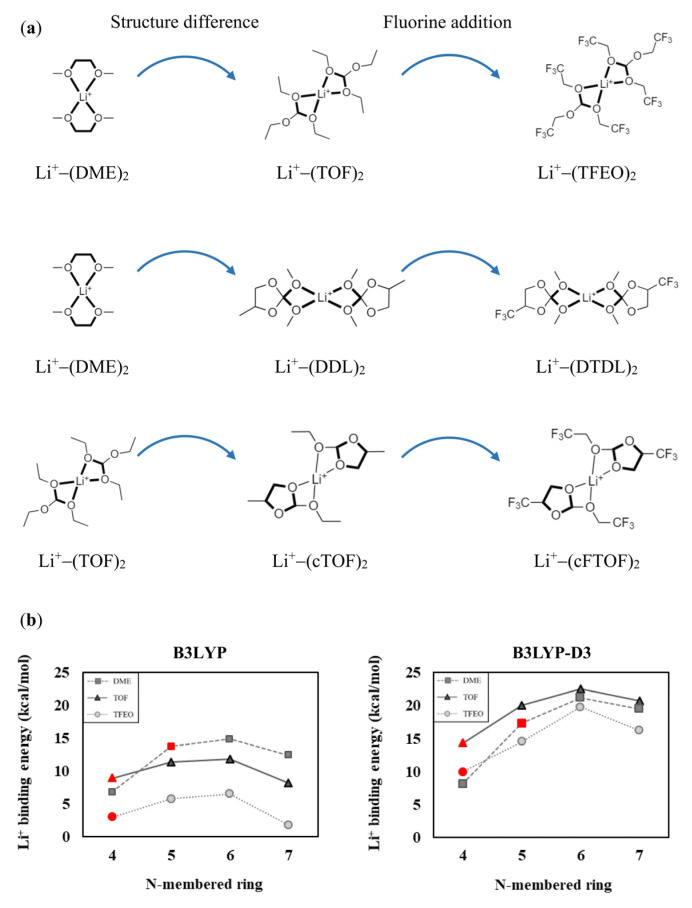

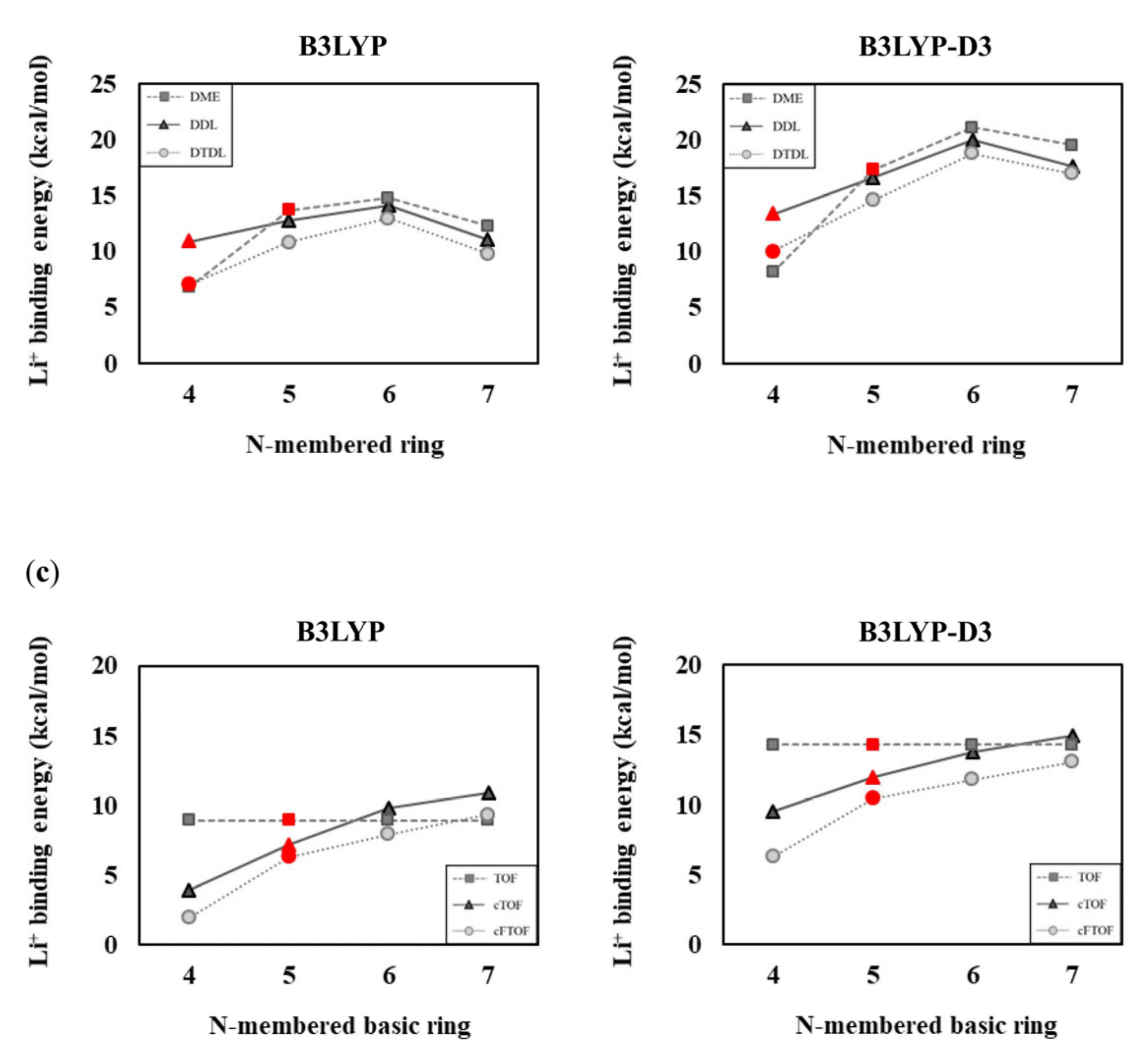
(**a**) Two solvent-coordinated Li^+^ solvation structures of DME, TOF, TFEO, DDL, DTDL, cTOF, and cFTOF molecules. Comparison of Li^+^ binding energies according to (**b**) the N-membered ring and (**c**) the N-membered basic ring. The DME, TOF, TFEO, DDL, DTDL, cTOF, and cFTOF molecules are highlighted in red. The Li^+^ binding energies are determined as E_b_ = −[E(Li^+^−(solvent)_2_ complex) − E(Li^+^) − 2 × E(solvent)]/2.

**Figure 7:**
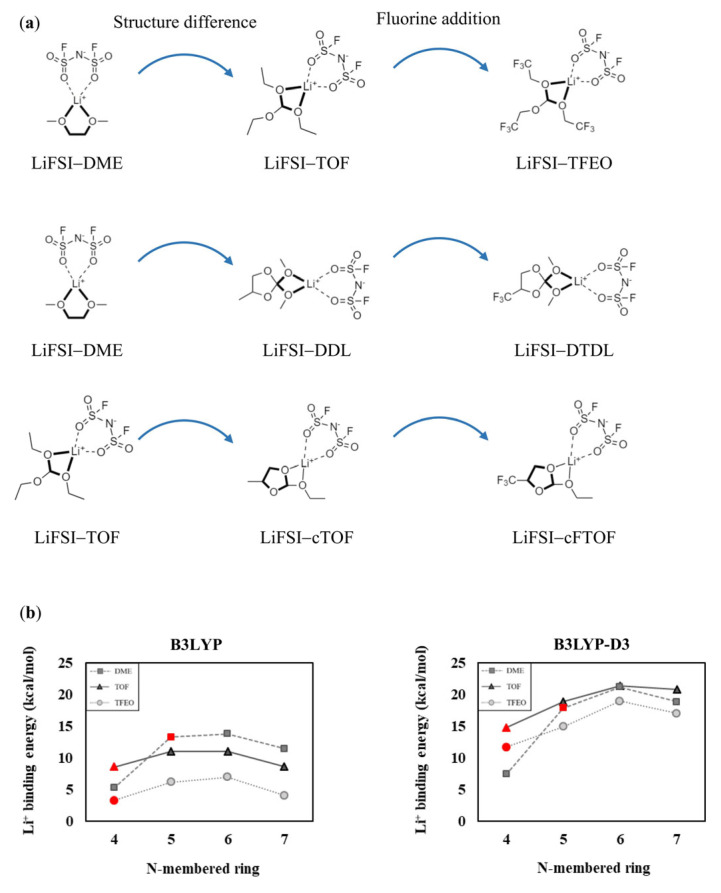

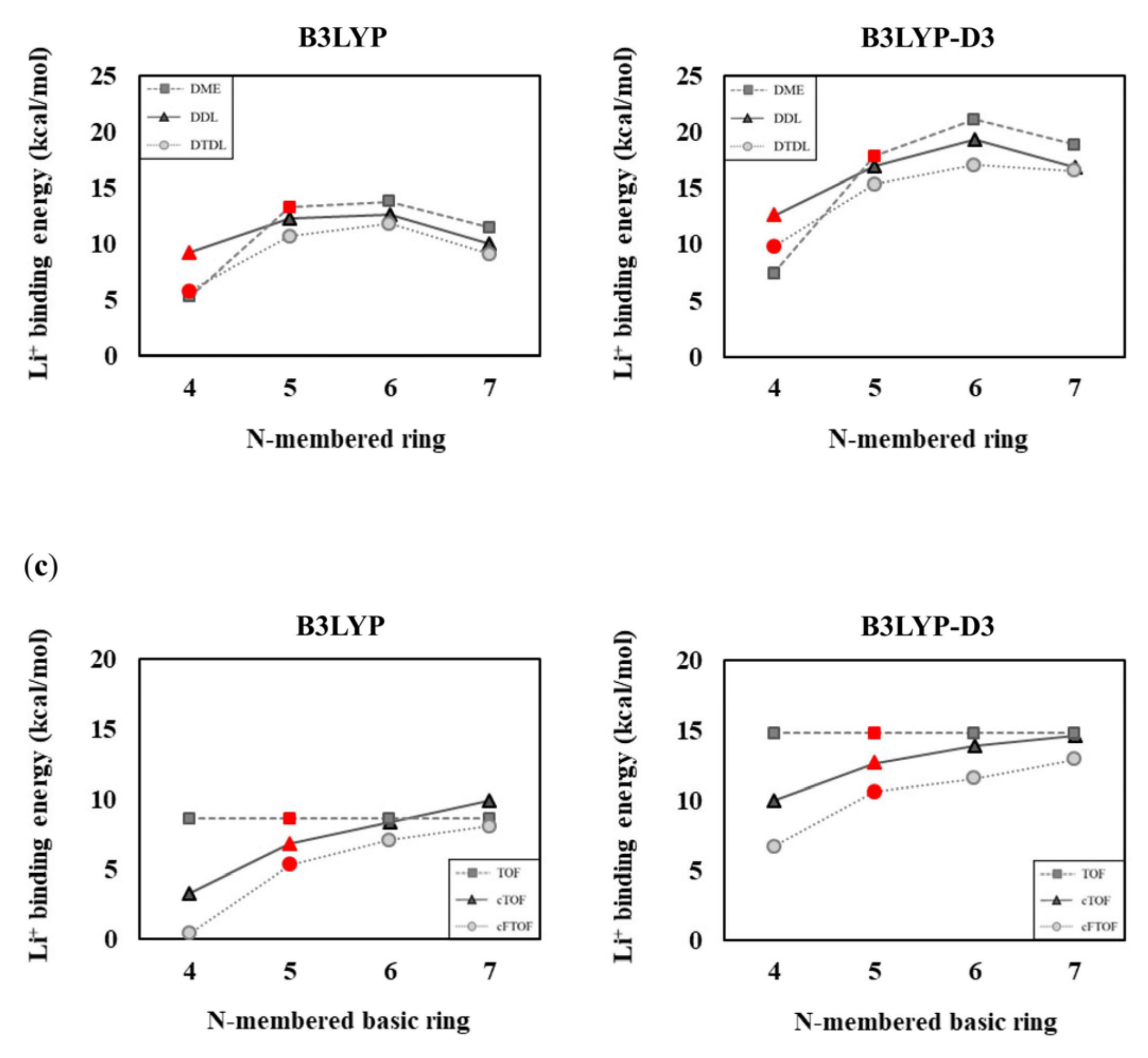
(**a**) One solvent and FSI anion-coordinated Li^+^ solvation structures of DME, TOF, TFEO, DDL, DTDL, cTOF, and cFTOF molecules. Comparison of Li^+^ binding energies according to the (**b**) N-membered ring and (**c**) N-membered basic ring. The DME, TOF, TFEO, DDL, DTDL, cTOF, and cFTOF molecules are highlighted in red. The Li^+^ binding energies are determined as E_b_ = −[E(LiFSI−solvent complex) − E(LiFSI) − E(solvent)].

In an actual electrolyte system, Li^+^ typically undergoes four-coordination with various solvents and/or salt anions to create a solvation sheath. Consequently, considering solvent−solvent and solvent−salt interactions (e.g., dipole−dipole interactions and dispersion interactions) is essential. We calculated the (i) binding energies of Li^+^ interacting with two solvents in each of the two-coordination modes (Figure 6) and (ii) binding energies of Li^+^ interacting with one solvent and one FSI anion in each of the two-coordination modes (Figure 7). We also included the B3LYP-D3 results with the B3LYP calculations in Figure 6 and Figure 7 to consider the dispersion interactions within the Li^+^ solvation sheath. All the calculated structures are presented in Figures S10 and S11.

The binding energy values are generally lower when two solvents interact with Li^+^ than when one solvent interacts with Li^+^. This is because Li^+^ is already stabilized through its interaction with one solvent; therefore, the interaction with the second solvent decreases. Our results also showed this, especially in cases where N = 6 and 7, where the binding energy decreases more significantly, as shown in Figure 6. This is interpreted as a steric effect between the large size of the solvents, and consequently, in the case of TOF and TFEO, the binding energy is lower at N = 7 than at N = 4. The B3LYP-D3 results show that the overall binding energy increased compared to the B3LYP results. This can be interpreted as reflecting the attractive dispersion interaction between solvents, and as a result, in the case of TOF and TFEO, the binding energy was higher at N = 7 than at N = 4. The calculated results for the Li^+^:solvent = 1:2 composition are consistent with the results in Section 3.1 and Section 3.2, confirming the validity of our assessment.

Similarly, in Figure 7, when one LiFSI is already attached and interacts with one solvent, the Li^+^−solvent binding energies are lower than when only one solvent interacts with Li^+^. Especially in the cases of N = 6 and 7, the binding energy decreases to a greater extent due to the increased instability caused by steric effects between the solvent and FSI^−^ moieties; however, the calculation results considering the salt remain consistent with the calculation results that do not consider the salt. The B3LYP-D3 values are higher than those obtained with B3LYP due to the dispersion interactions occurring between the FSI anion and the solvent. However, the trend in Li^+^ binding energies with respect to the N values remains consistent with the results for the 1:1 and 1:2 compositions, as shown in Figure 7. In summary, when considering solvent−solvent and solvent−salt interactions, the influence of structural strain from N-membered rings or basic rings on Li^+^ binding energies appears consistent, regardless of the computational structure models. Specifically, as the ring size increases from a four-membered to a six-membered ring, the ring strain decreases, resulting in an increase in Li^+^ binding energy. However, in the case of a seven-membered ring, torsional strain causes a decrease in the binding energy. In an N-membered basic ring, as the value of N increases, the structural strain decreases, leading to an increase in Li^+^ binding energy.

The rings formed by the interaction between the oxygen-binding site and Li^+^ and the rings within the molecule containing the oxygen-binding site can have a significant impact on the interaction with Li^+^. This phenomenon can be analyzed by interpreting the structural strain and Li^+^ binding energy. If the oxygen-binding site undergoes less structural strain, it can ensure a structure suitable for favorable interaction with Li^+^ ions, resulting in higher Li^+^ binding energy, while the opposite occurs if it undergoes more structural strain. Our computational results show a clear trend in the interpretation of the solvation ability of various solvent molecules and can be highly effective in interpreting the experimental results. Moreover, experimental studies on the solvation ability of BTFM (bis(2,2,2-trifluoroethoxy)methane) [92], DMM (dimethoxymethane) [93,94], and DMP (1,3-dimethoxypropane) [95] have reported results that could be interpreted as in our calculations. Both BTFM and DMM interacting with Li^+^ showed a lower solvation ability than DME due to the high structural strain of the four-membered ring. On the other hand, DMP, which forms a six-membered ring by interacting with Li^+^, showed a higher solvation ability than DME, with a low structural strain.

To analyze whether the nitrogen atoms, when utilized as binding sites, contribute to the solvation ability in a manner similar to oxygen, we substituted O atoms with NH in the structures of four- and six-membered rings and four- and seven-membered basic rings, and calculated the Li^+^ binding energy. As shown in Figure S12, similar to the case of oxygen, the binding energy in the six-membered ring is greater than in the four-membered ring, and the binding energy in the seven-membered basic ring is greater than in the four-membered basic ring. This suggests that even if the element coordinating Li^+^ is changed from O to another element, there will be a change in binding energy due to ring strain. Therefore, we believe that improving the understanding of these structural characteristics can significantly contribute to understanding the solvation ability of organic solvent molecules and to control the Li^+^ solvation sheath at the molecular level more effectively.

## 4. Conclusions

We theoretically analyzed how solvation ability can be controlled to improve battery performance using newly developed organic solvents with excellent performance: TFEO, DTDL, and cFTOF. The decrease in Li^+^ solvation ability of TFEO, DTDL, and cFTOF compared to DME and TOF is influenced not only by the effect of fluorine substitution but also by the structural characteristics. When an N-membered ring is formed by the interaction between Li^+^ and organic molecules, if the size of the N-membered ring is small, the structural strain of the molecule increases due to increased ring strain, and Li^+^ binding energy decreases. In addition, the basic ring containing the oxygen binding site present in the organic solvent itself also affects the Li^+^ binding energy. If the oxygen binding site is included in a four- or five-membered basic ring, the oxygen binding site cannot secure a suitable structure for orbital interaction with Li^+^ due to the small internal angle of the basic ring, leading to a decreased solvation ability. We have improved our understanding of the solvation ability of organic solvents by demonstrating that structural factors significantly contribute to the Li^+^ solvation ability of solvents. We suggest that considering both the electrical and structural characteristics of electrolyte solvents is a practical and effective approach when attempting to regulate the interaction between organic solvents and metal cations.

## Data Availability

The data used to support the findings of this study are available from the corresponding author upon request.

## Supplementary Materials

The following supporting information can be downloaded at https://www.mdpi.com/article/10.3390/ma16216995/s1, Figure S1: Optimized structures of (a) DME, TOF, and TFEO, (b) DME, DDL, and DTDL, and (c) TOF, cTOF, and cFTOF; Figure S2: One- and two-coordinate binding energies between lithium ion and organic solvent, and ring formation energy; Figure S3: Optimized structures of (a) DME, TOF, and TFEO, (b) DME, DDL, and DTDL, and (c) TOF, cTOF, and cFTOF coordinated to Li^+^; Figure S4: Li^+^-coordinated structures of 4- to 7-membered 2D ring structures based on DME, TOF, TFEO, DDL, and DTDL molecules; Figure S5: Comparison of one-coordinate (E_b1_) and two-coordinate (E_b2_) binding energies of 4- to 7-membered ring structures. The ring formation energy (E_R_) is the reaction energy when Li^+^ is converted from a one-coordinate to a two-coordinate form with the solvent molecules. Units are in kcal/mol; Figure S6: Second-order perturbation theory analysis through NBO analysis in (a) 4-membered ring and (b) 6-membered ring; Figure S7: Contour plots of oxygen lone pair orbitals and lithium antibonding lone pair orbitals of TOF, cTOF, and cFTOF coordinated to Li^+^. The black arrows represent the direction of lone pair orbitals of the oxygen binding sites. The bold red lines are the 0.020 isovalue lines of oxygen lone pair orbitals; Figure S8: Second order perturbation theory analysis through NBO analysis in (a) 4-membered basic ring and (b) 7-membered basic ring; Figure S9: Calculated Li^+^ binding energies according to the N of (a) DME, TOF, TFEO, (b) DME, DDL, DTDL, and (c) TOF, cTOF, and cFTOF at the B3LYP-D3, M05-2X, PBE, and M06-L levels of theory. The DME, TOF, TFEO, DDL, DTDL, cTOF, and cFTOF results are highlighted in red; Figure S10: Two solvent-coordinated Li^+^ structures of 4- to 7-membered 2D ring structures based on (a) DME, TOF, TFEO, DDL, and DTDL, and (b) cTOF and cFTOF molecules; Figure S11: One solvent and FSI anion-coordinated Li^+^ solvation structures of 4- to 7-membered 2D ring structures based on (a) DME, TOF, TFEO, DDL, and DTDL and (b) cTOF and cFTOF molecules; Figure S12: 2D structures and calculated Li^+^ binding energies of the Li^+^ coordinated solvent molecules where oxygens are substituted with NH groups. Units are in kcal/mol; Table S1: The calculated Li−O−C bond angles, ring strain energies, and Li^+^ binding energies of 4- to 7-membered rings based on (a) DME, TOF, and TFEO and (b) DME, DDL, and DTDL; Table S2: The calculated values of one- (E_b1_) and two-coordinated (E_b2_) Li^+^ binding energies, ring formation energies (E_R_) of 4- to 7-membered rings based on (a) DME, TOF, and TFEO and (b) DME, DDL, and DTDL. Units are in kcal/mol; Table S3: The calculated Li−O−C bond angles and Li^+^ binding energies of 4- to 7-membered basic rings based on TOF, cTOF, and cFTOF.
