# Supplementary material for: Origin of Li+ Solvation Ability of Electrolyte Solvent: Ring Strain
Source: Materials (Basel). 2023 Oct 31;16(21):6995. doi: 10.3390/ma16216995 (PMC10650738; doi:10.3390/ma16216995)
Supplement: Supplementary file 1 [file materials-16-06995-s001.zip › materials-2684446-supplementary.pdf]

Supplementary data for

Origin of Li+ Solvation Ability of Electrolyte Solvent: Ring Strain

Jihoon Choi [1], Kyoung-Hee Shin [2] and Young-Kyu Han [1,]*

1 Department of Energy and Materials Engineering, Advanced Energy and Electronic Materials Research Center, Dongguk University-Seoul, Seoul 04620, Republic of Korea; chchc2059@gmail.com

2 ESS Laboratory, Korea Institute of Energy Research, 102 Gajeong-ro, Daejeon 34129, Republic of Korea; khshin@kier.re.kr

* Correspondence: ykenergy@dongguk.edu

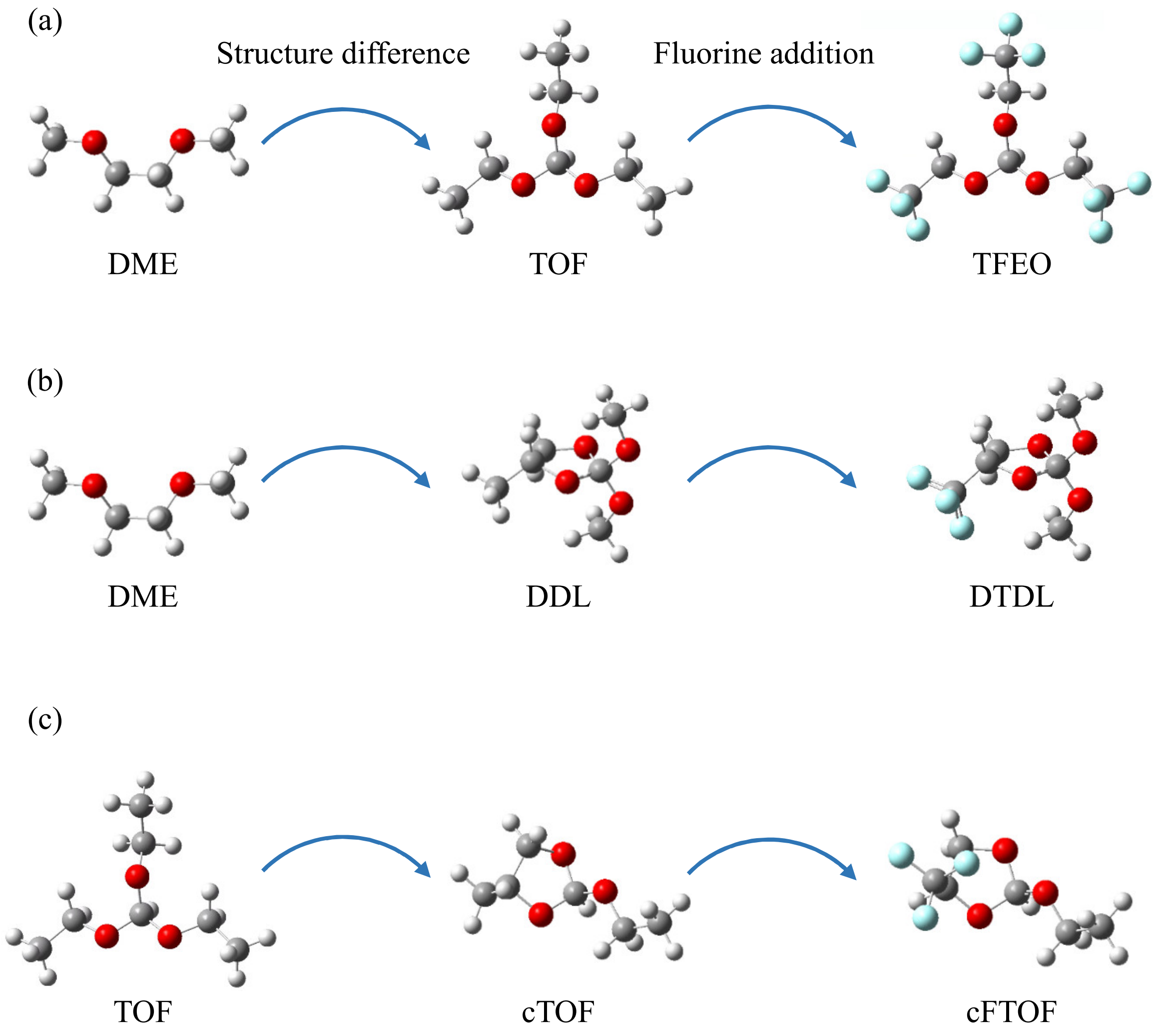

Figure S1. Optimized structures of (a) DME, TOF, and TFEO, (b) DME, DDL, and DTDL, and (c) TOF, cTOF, and cFTOF.

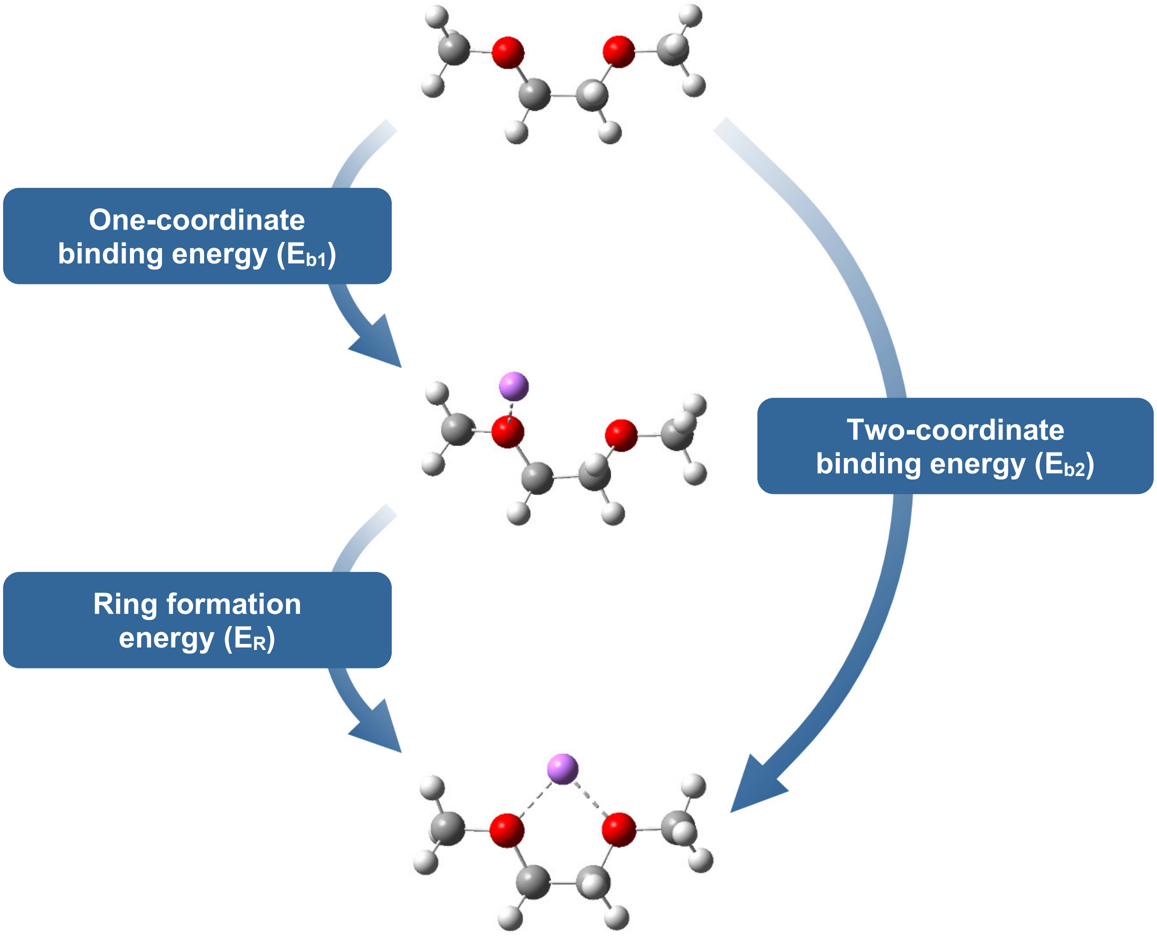

Figure S2. One- and two-coordinate binding energies between lithium ion and organic solvent, and ring formation energy.

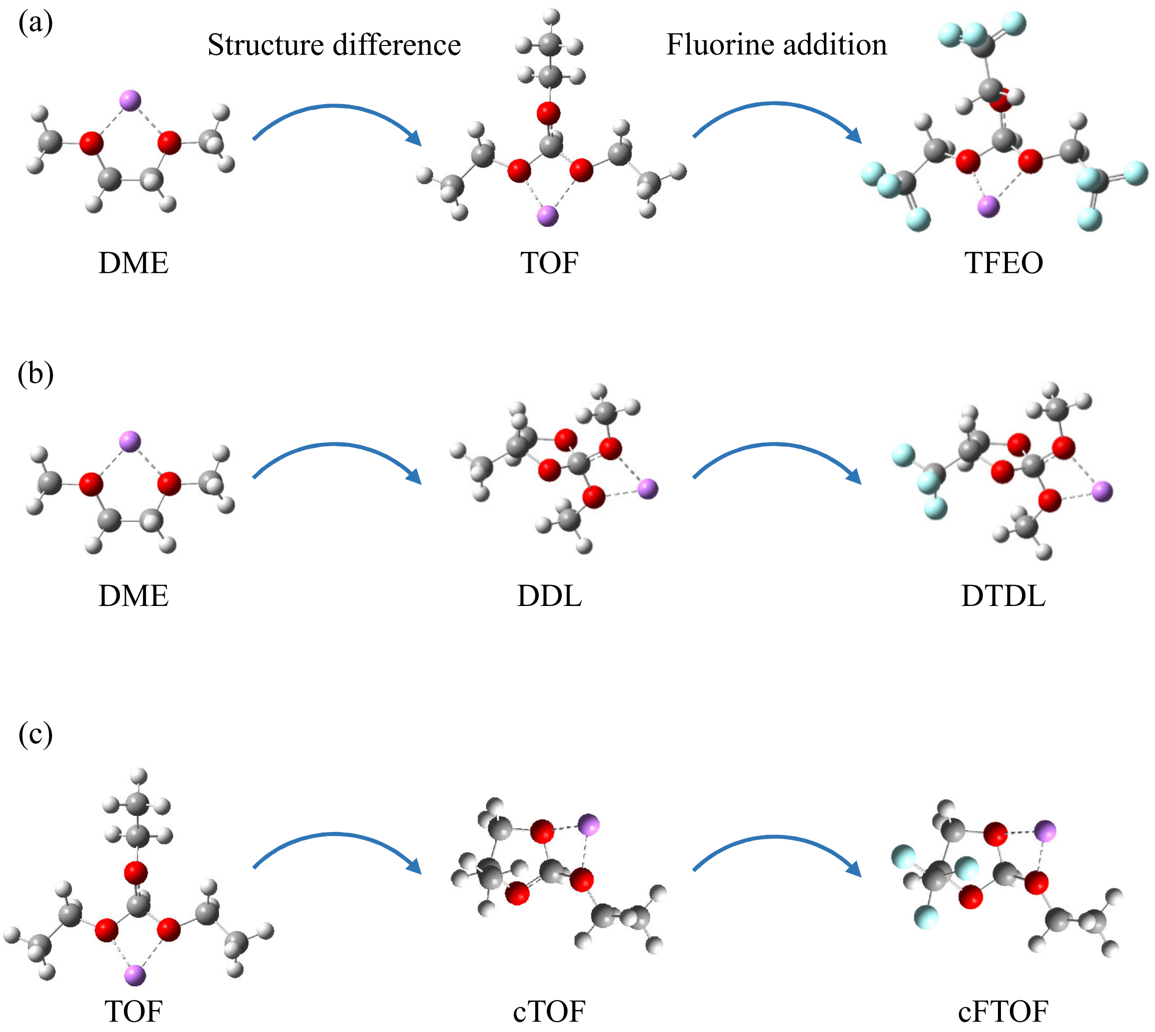

Figure S3. Optimized structures of (a) DME, TOF, and TFEO, (b) DME, DDL, and DTDL, and (c) TOF, cTOF, and cFTOF coordinated to Li^+.

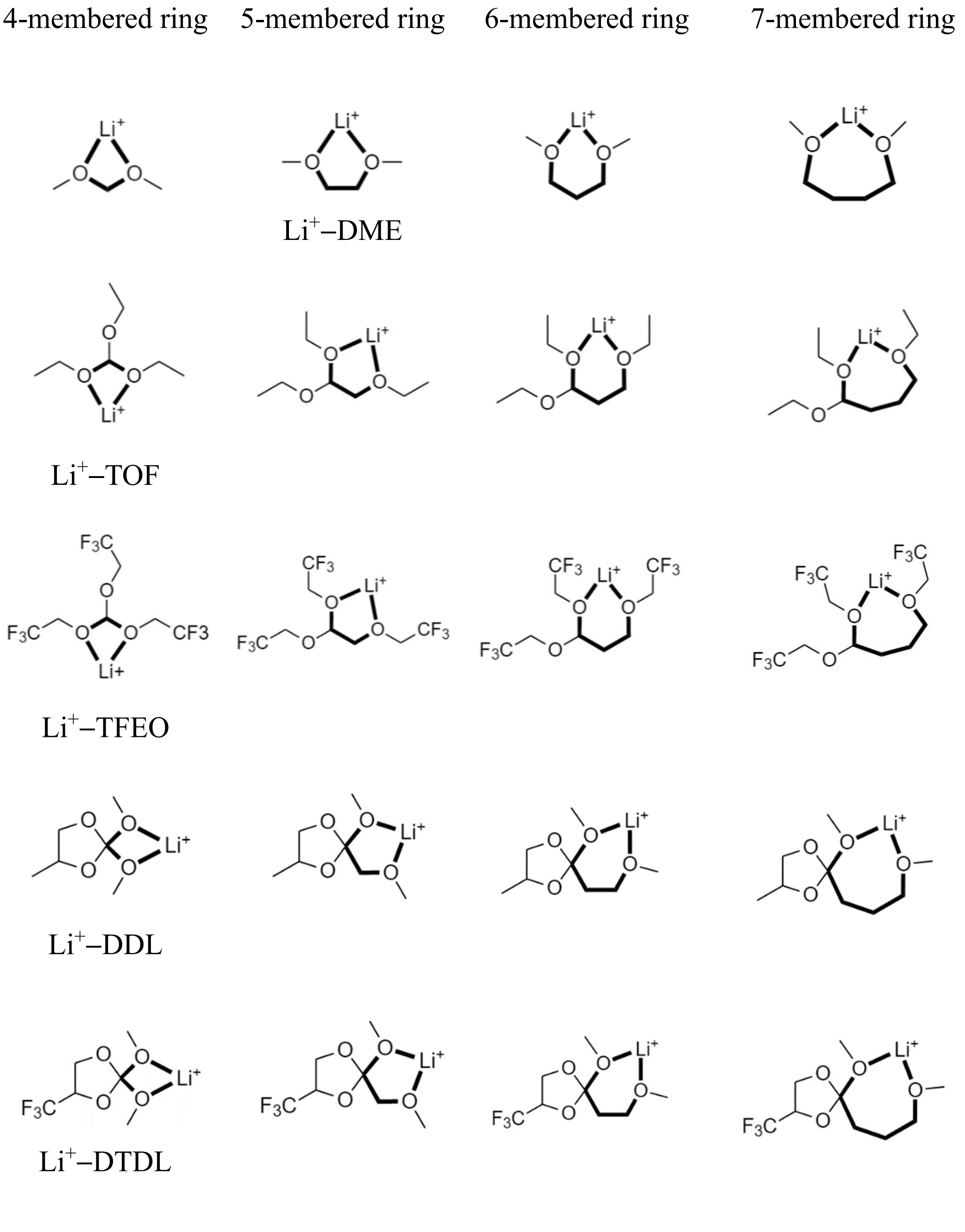

Figure S4. Li$^+$-coordinated structures of 4- to 7-membered 2D ring structures based on DME, TOF, TFEO, DDL, and DTDL molecules.

Table S1. The calculated Li–O–C bond angles, ring strain energies, and Li^+ binding energies of 4- to 7-membered ring based on (a) DME, TOF, and TFEO and (b) DME, DDL, and DTDL.

(a)

N-membered ring		N = 4	N = 5	N = 6	N = 7
Li–O–C bond angle (θ in degree)	DME	97.0	108.8	122.2	123.8
	TOF	97.3	110.7	119.8	122.0
	TFEO	99.1	111.7	118.0	123.2
Ring strain (kcal/mol)	DME	7.6	1.3	0.0	2.6
	TOF	3.6	0.7	0.0	2.3
	TFEO	2.7	0.3	0.0	1.4
Li^+ binding energy (kcal/mol)	DME	8.3	15.6	17.2	14.9
	TOF	11.5	14.4	15.1	12.7
	TFEO	3.6	6.3	6.9	5.7

(b)

N-membered ring		N = 4	N = 5	N = 6	N = 7
Li–O–C bond angle (θ in degree)	DME	97.0	108.8	122.2	123.8
	DDL	98.3	112.6	121.9	125.3
	DTDL	98.2	112.5	122.8	127.3
Ring strain (kcal/mol)	DME	7.6	1.3	0.0	2.6
	DDL	3.1	1.1	0.0	2.5
	DTDL	4.9	1.6	0.0	3.2
Li^+ binding energy (kcal/mol)	DME	8.3	15.6	17.2	14.9
	DDL	12.5	14.3	15.6	13.3
	DTDL	8.2	12.4	14.5	11.7

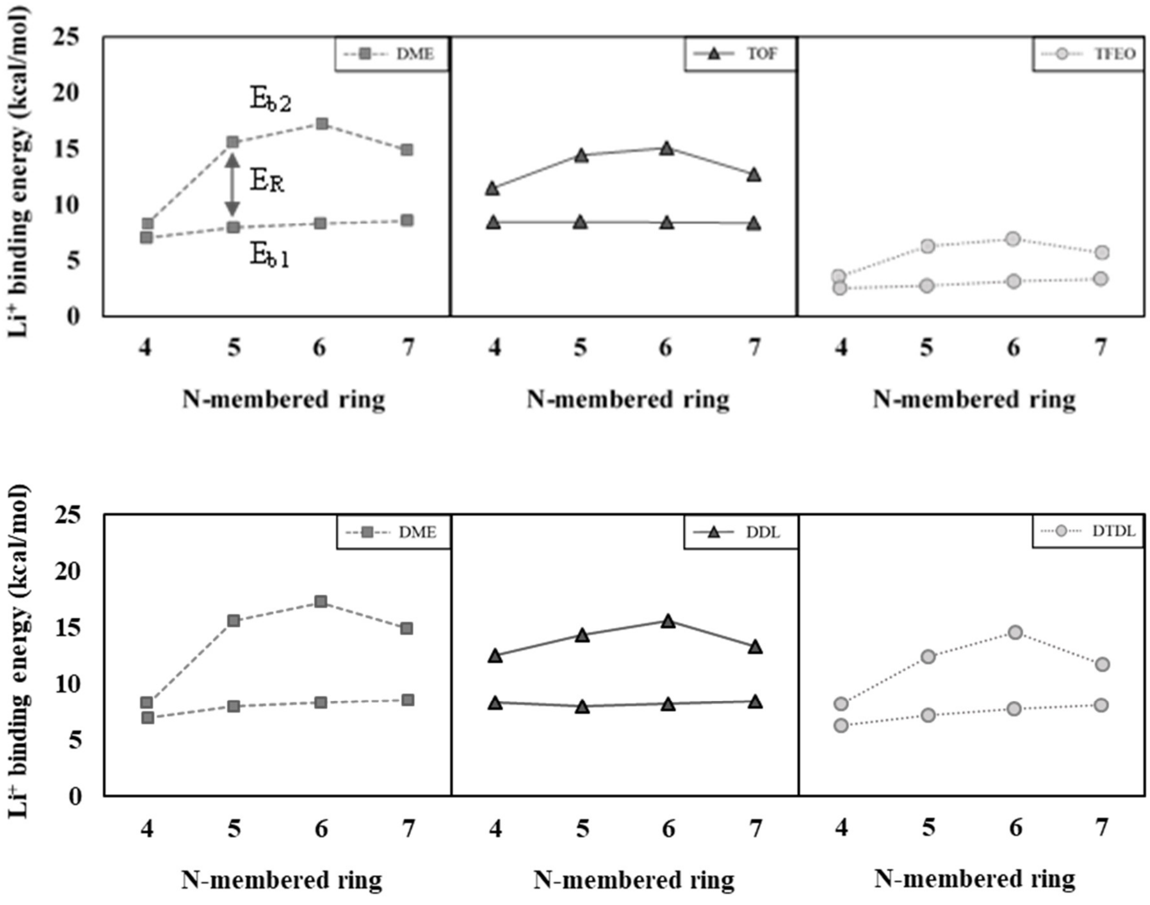

Figure S5. Comparison of one-coordinate (E_{b1}) and two-coordinate (E_{b2}) binding energies of 4- to 7-membered ring structures. The ring formation energy (E_R) is the reaction energy when Li^+ is converted from a one-coordinate to a two-coordinate form with the solvent molecules. Units are in kcal/mol.

Table S2. The calculated values of one (E_{b1}) and two-coordinated (E_{b2}) Li^+ binding energies, ring formation energies (E_R) of 4- to 7-membered ring based on (a) DME, TOF, and TFEO and (b) DME, DDL, and DTDL. Units are in kcal/mol.

(a)

N-membered ring		N = 4	N = 5	N = 6	N = 7
DME	E_{b1}	7.0	7.9	8.3	8.6
	E_R	1.3	7.7	8.9	6.3
	E_{b2}	8.3	15.6	17.2	14.9
TOF	E_{b1}	8.5	8.5	8.4	8.4
	E_R	3.0	5.9	6.7	4.3
	E_{b2}	11.5	14.4	15.1	12.7
TFEO	E_{b1}	2.5	2.8	3.1	3.3
	E_R	1.1	3.5	3.8	2.4
	E_{b2}	3.6	6.3	6.9	5.7

(b)

N-membered ring		N = 4	N = 5	N = 6	N = 7
DME	E_{b1}	7.0	7.9	8.3	8.6
	E_R	1.3	7.7	8.9	6.3
	E_{b2}	8.3	15.6	17.2	14.9
DDL	E_{b1}	8.3	8.0	8.3	8.5
	E_R	4.2	6.3	7.3	4.8
	E_{b2}	12.5	14.3	15.6	13.3
DTDL	E_{b1}	6.2	7.2	7.7	8.1
	E_R	1.9	5.2	6.8	3.6
	E_{b2}	8.2	12.4	14.5	11.7

(a)

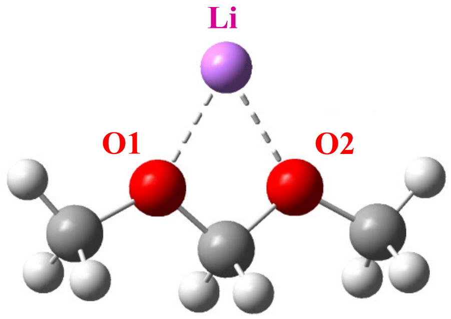

Donor (i)	Acceptor (j)	E2[a] (kcal/mol)	E(j) – E(i)[b] (a.u.)	F(i,j)[c] (a.u.)
LP (1) O1	LP* (1) Li	4.92	0.77	0.06
	LP* (3) Li	2.41	0.76	0.04
LP (1) O2	LP* (1) Li	4.92	0.77	0.06
	LP* (3) Li	2.41	0.76	0.04

[a] E2 represent the stabilization energy.
[b] The energy difference between donor (i) and acceptor (j) orbitals.
[c] The interaction element between donor (i) and acceptor (j) orbitals.

(b)

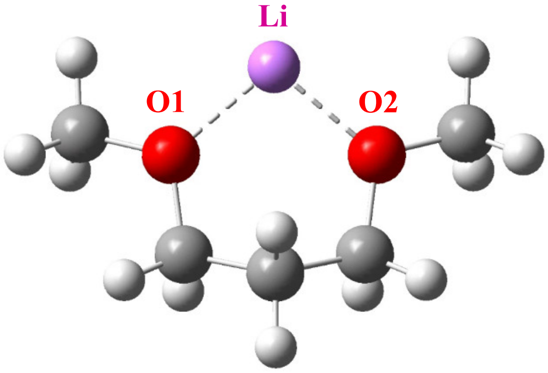

Donor (i)	Acceptor (j)	E2 (kcal/mol)	E(j) – E(i) (a.u.)	F(i,j) (a.u.)
LP (1) O1	LP* (1) Li	7.23	0.78	0.07
	LP* (2) Li	4.52	0.76	0.05
LP (1) O2	LP* (1) Li	7.23	0.78	0.07
	LP* (2) Li	4.52	0.76	0.05

Figure S6. Second order perturbation theory analysis through NBO analysis in (a) 4-membered ring and (b) 6-membered ring.

Table S3. The calculated Li−O−C bond angles and Li^+ binding energies of 4- to 7-membered basic ring based on TOF, cTOF, and cFTOF.

N-membered basic ring		N = 4	N = 5	N = 6	N = 7
Li−O−C bond angle (θ in degree)	TOF	97.3	97.3	97.3	97.3
	cTOF	94.5	95.5	95.9	96.9
	cFTOF	94.5	95.8	96.1	97.2
Li^+ binding energy (kcal/mol)	TOF	11.5	11.5	11.5	11.5
	cTOF	5.5	8.8	11.7	13.2
	cFTOF	2.1	7.6	9.6	11.2

TOF: Low structural strain

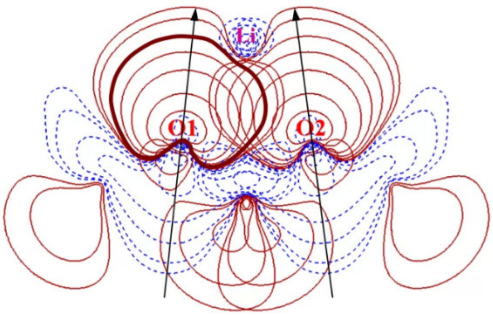

cTOF: High structural strain

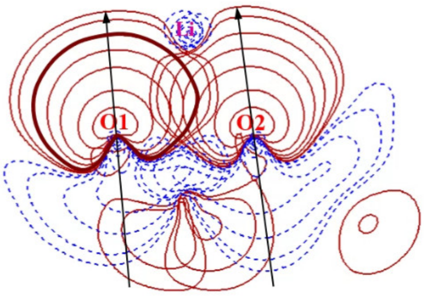

cFTOF: High structural strain

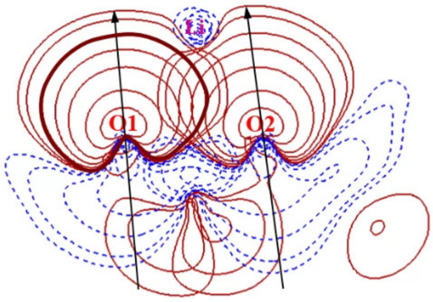

Figure S7. Contour plots of oxygen lone pair orbitals and lithium antibonding lone pair orbitals of TOF, cTOF, and cFTOF coordinated to Li^+. The black arrows represent the direction of lone pair orbitals of the oxygen binding sites. The bold red lines are the 0.020 isovalue lines of oxygen lone pair orbitals.

(a)

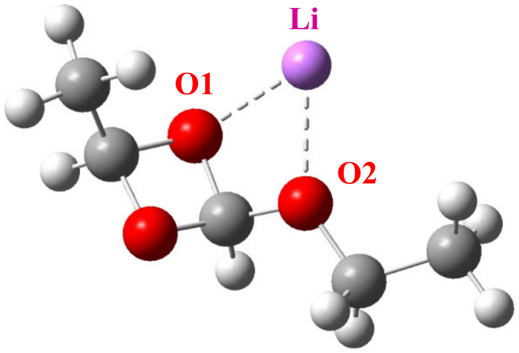

Donor (i)	Acceptor (j)	E2 (kcal/mol)	E(j) – E(i) (a.u.)	F(i,j) (a.u.)
LP (1) O1	LP* (1) Li	3.28	0.81	0.05
	LP* (2) Li	3.45	0.79	0.05
LP (1) O2	LP* (1) Li	4.85	0.72	0.05
	LP* (2) Li	1.43	0.70	0.03

(b)

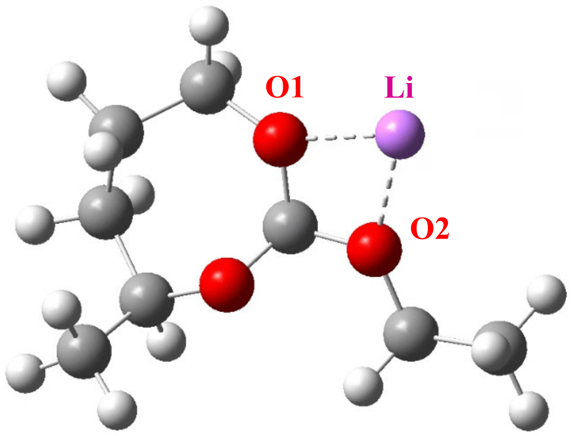

Donor (i)	Acceptor (j)	E2 (kcal/mol)	E(j) – E(i) (a.u.)	F(i,j) (a.u.)
LP (1) O1	LP* (1) Li	4.03	0.74	0.05
	LP* (2) Li	2.66	0.74	0.04
LP (1) O2	LP* (1) Li	5.64	0.75	0.06
	LP* (2) Li	1.89	0.74	0.03

Figure S8. Second order perturbation theory analysis through NBO analysis in (a) 4-membered basic ring and (b) 7-membered basic ring.

(a)

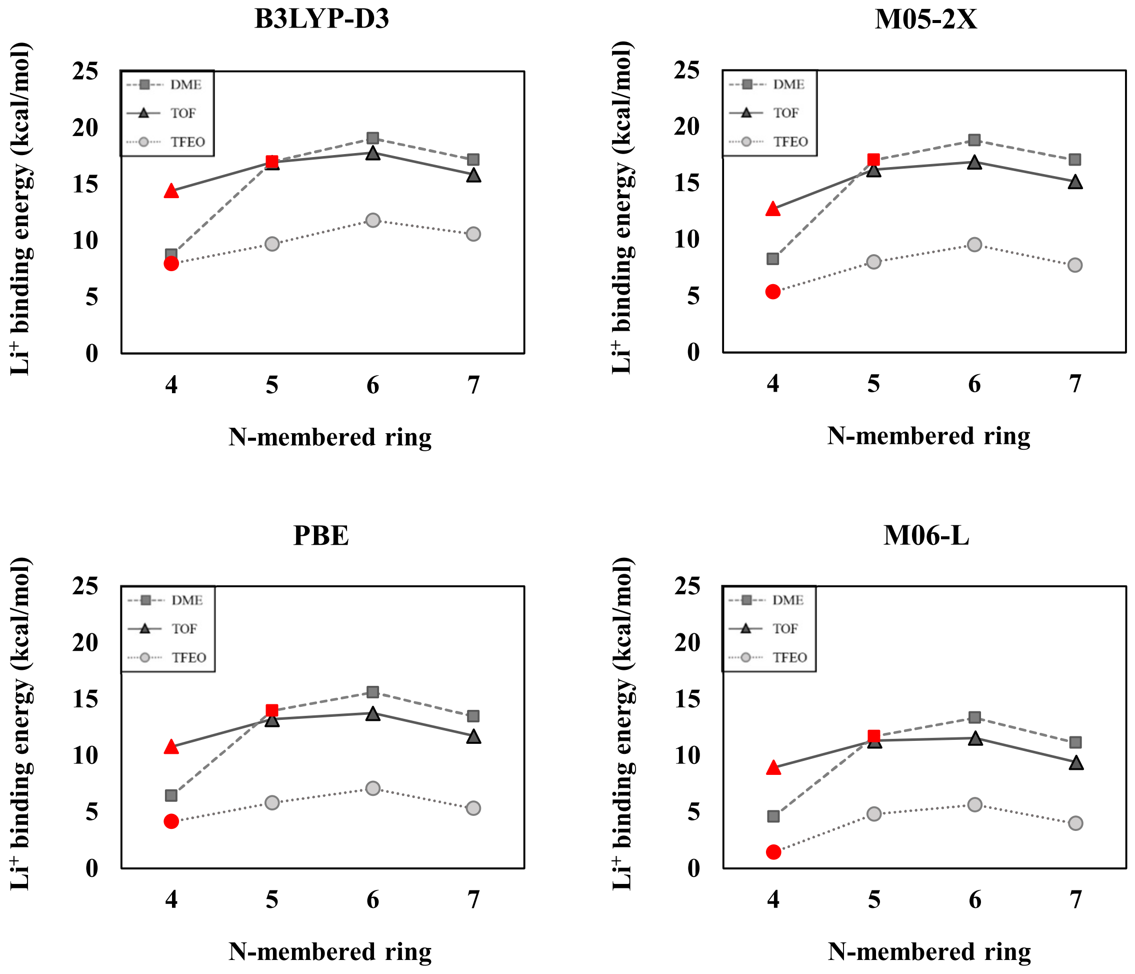
B3LYP-D3
M05-2X
PBE
M06-L
DME
TOF
TFEO
Li+ binding energy (kcal/mol)
N-membered ring
0
5
10
15
20
25
4
5
6
7

(b)

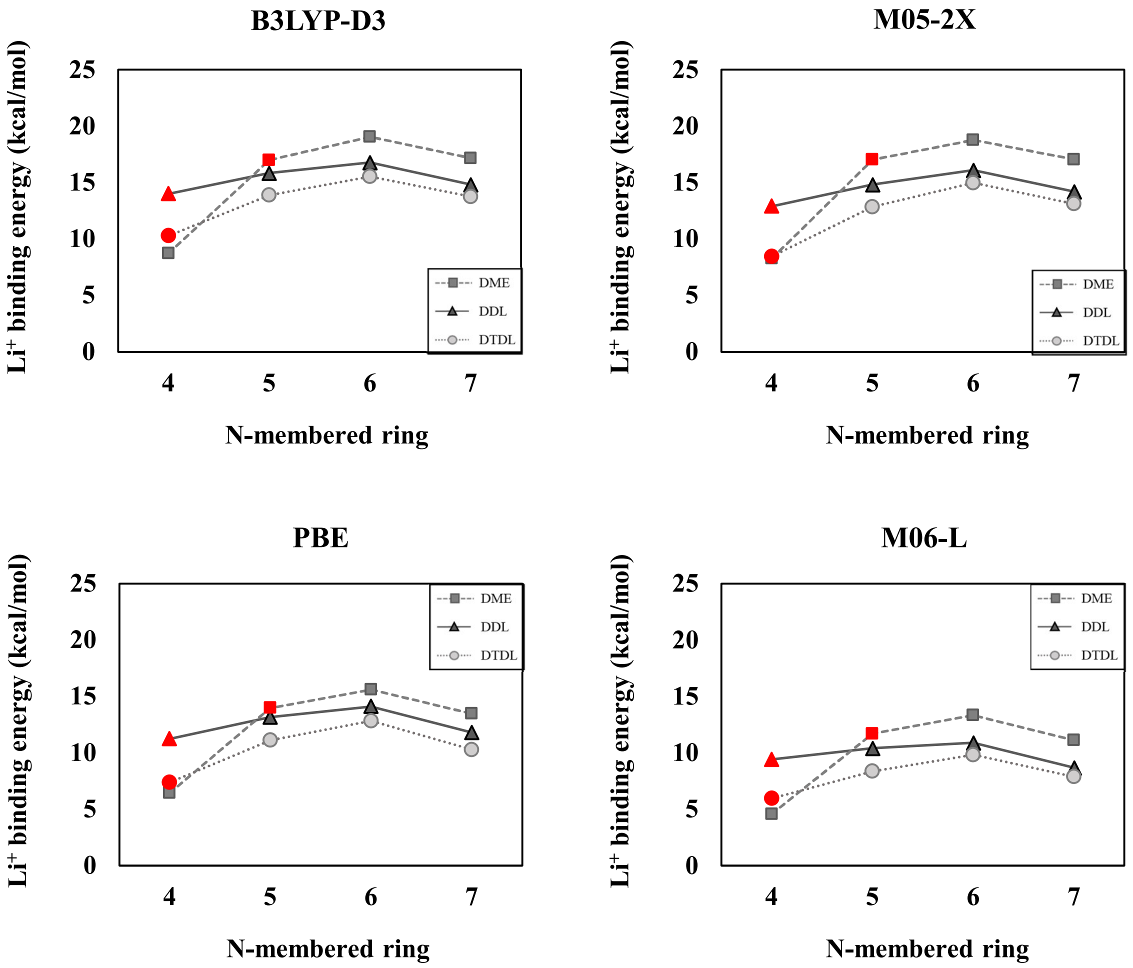
B3LYP-D3
M05-2X
PBE
M06-L
Li+ binding energy (kcal/mol)
N-membered ring
DME
DDL
DTDL
0
5
10
15
20
25
4
5
6
7

(c)

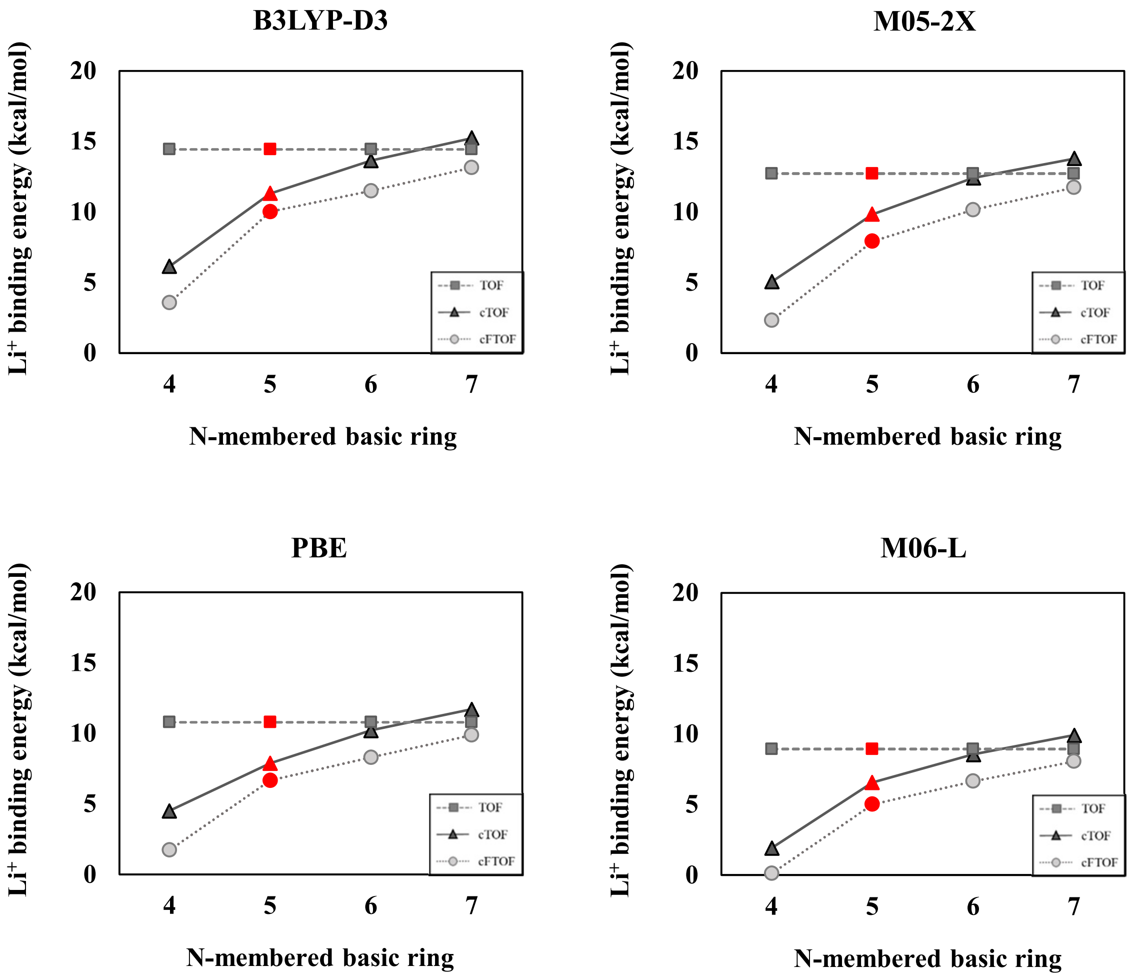

Figure S9. Calculated Li^+ binding energies according to the N of (a) DME, TOF, TFEO, (b) DME, DDL, DTDL, and (c) TOF, cTOF, and cFTOF at the B3LYP-D3, M05-2X, PBE, and M06-L levels of theory. The DME, TOF, TFEO, DDL, DTDL, cTOF, and cFTOF results are highlighted in red.

(a)

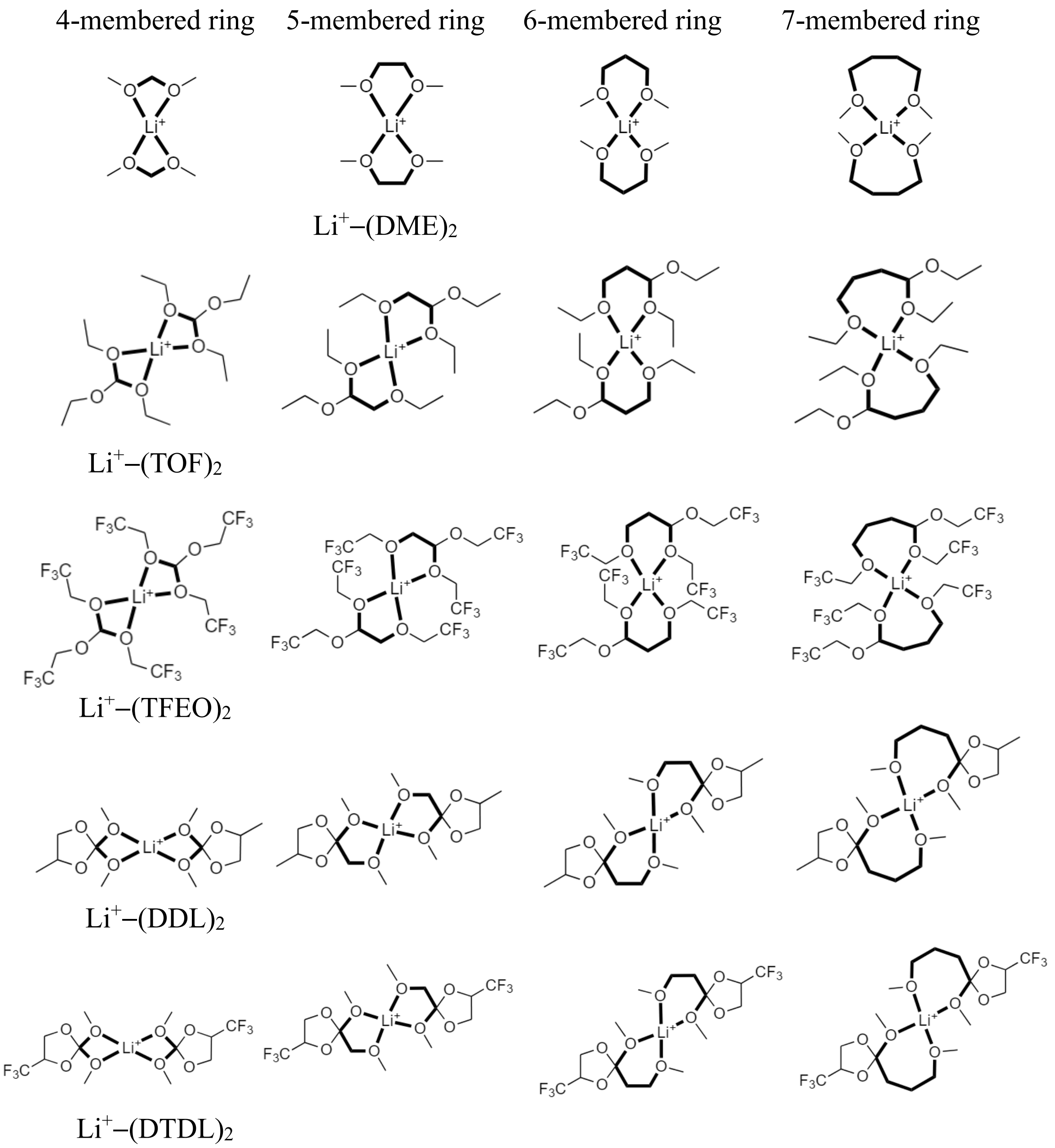
4-membered ring
5-membered ring
6-membered ring
7-membered ring
Li+–(DME)2
Li+–(TOF)2
Li+–(TFEO)2
Li+–(DDL)2
Li+–(DTDL)2

(b)

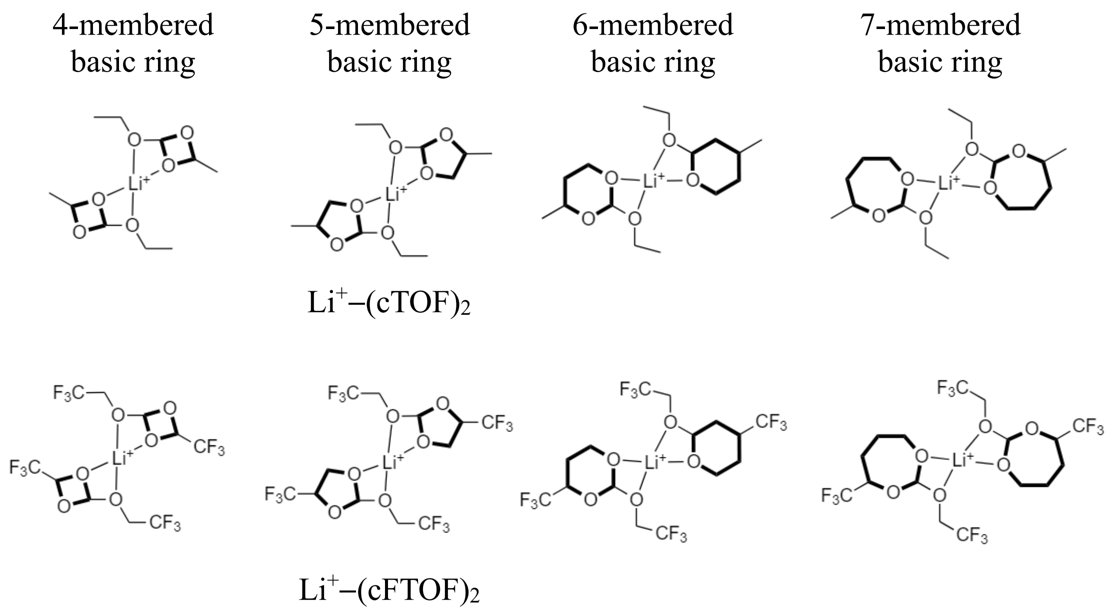

Figure S10. Two solvent-coordinated Li^+ structures of 4- to 7-membered 2D ring structures based on (a) DME, TOF, TFEO, DDL, and DTDL, and (b) cTOF and cFTOF molecules.

(a)

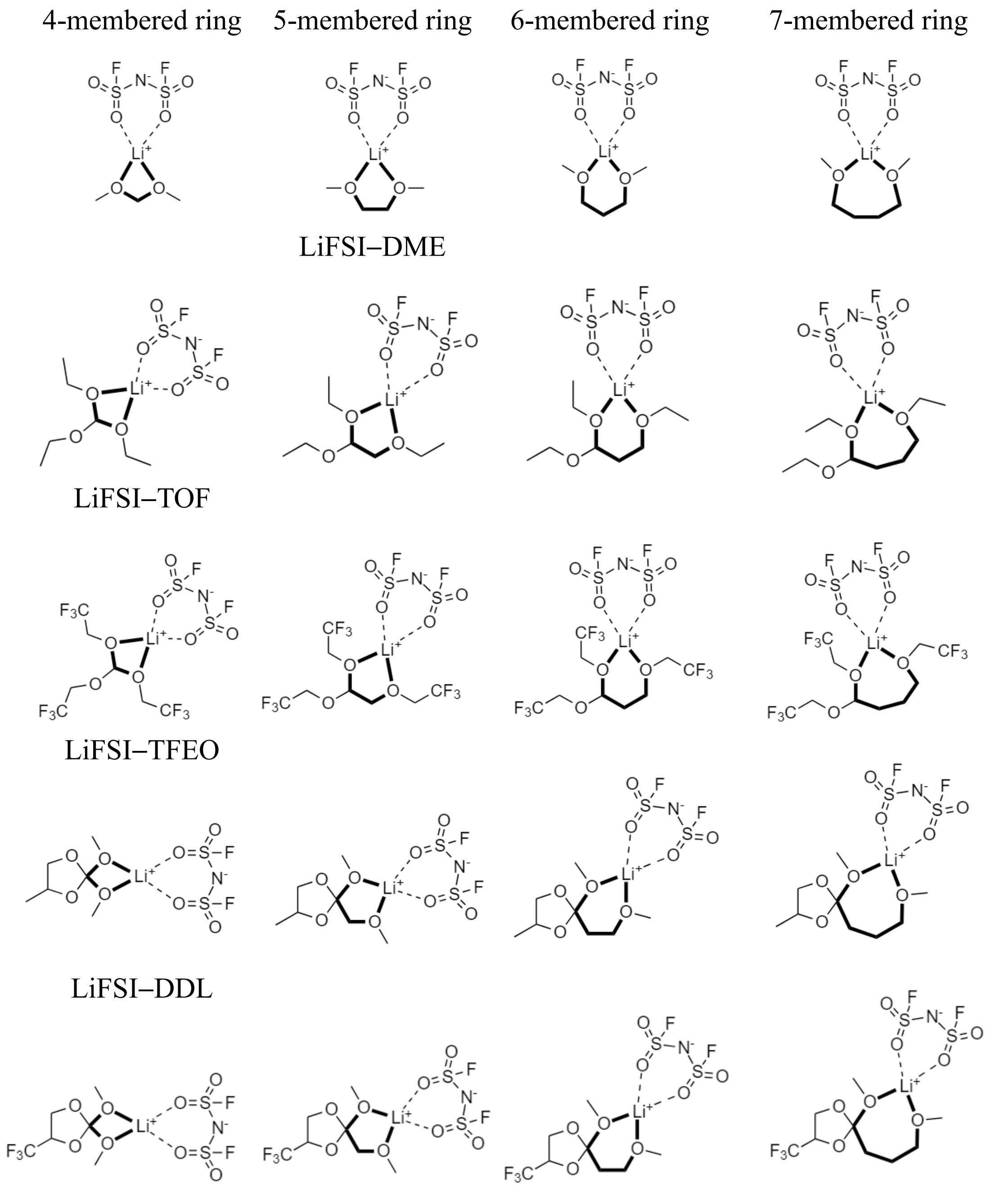
4-membered ring
5-membered ring
6-membered ring
7-membered ring
LiFSI–DME
LiFSI–TOF
LiFSI–TFEO
LiFSI–DDL
LiFSI–DTDL

(b)

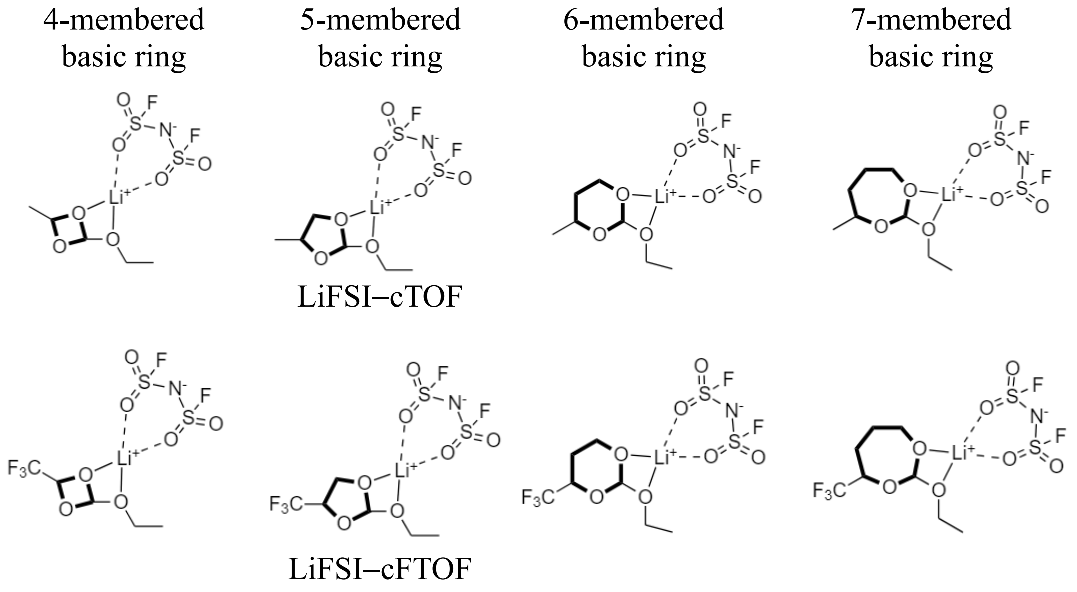

Figure S11. One solvent and FSI anion-coordinated Li^+ solvation structures of 4- to 7-membered 2D ring structures based on (a) DME, TOF, TFEO, DDL, and DTDL and (b) cTOF and cFTOF molecules.

4-membered ring: 13.4

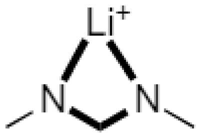

6-membered ring: 19.2

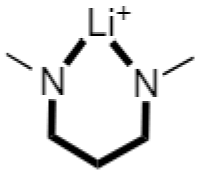

4-membered basic ring: 13.1

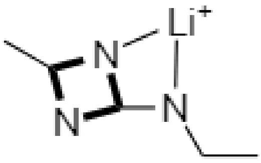

7-membered basic ring: 14.1

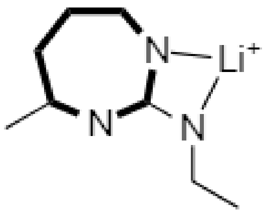

Figure S12. 2D structures and calculated Li^+ binding energies of the Li^+ coordinated solvent molecules where oxygens are substituted with NH groups. Units are in kcal/mol.
